# Lactate increases stemness of CD8 + T cells to augment anti-tumor immunity

**DOI:** 10.1038/s41467-022-32521-8

**Published:** 2022-09-06

**Authors:** Qiang Feng, Zhida Liu, Xuexin Yu, Tongyi Huang, Jiahui Chen, Jian Wang, Jonathan Wilhelm, Suxin Li, Jiwon Song, Wei Li, Zhichen Sun, Baran D. Sumer, Bo Li, Yang-Xin Fu, Jinming Gao

**Affiliations:** 1 Department of Pharmacology, Harold C. Simmons Comprehensive Cancer Center, University of Texas Southwestern Medical Center, Dallas, TX 75390 USA; 2 Department of Pathology, University of Texas Southwestern Medical Center, Dallas, TX 75390 USA; 3 Lyda Hill Department of Bioinformatics, University of Texas Southwestern Medical Center, Dallas, TX 75390 USA; 4 Department of Otolaryngology, University of Texas Southwestern Medical Center, Dallas, TX 75390 USA; 5 Department of Immunology, University of Texas Southwestern Medical Center, Dallas, TX 75390 USA; 6 Department of Cell Biology, University of Texas Southwestern Medical Center, Dallas, TX 75390 USA

**Keywords:** Tumour immunology, Immunotherapy, Lymphocyte activation, Cancer metabolism

## Abstract

Lactate is a key metabolite produced from glycolytic metabolism of glucose molecules, yet it also serves as a primary carbon fuel source for many cell types. In the tumor-immune microenvironment, effect of lactate on cancer and immune cells can be highly complex and hard to decipher, which is further confounded by acidic protons, a co-product of glycolysis. Here we show that lactate is able to increase stemness of CD8^+^ T cells and augments anti-tumor immunity. Subcutaneous administration of sodium lactate but not glucose to mice bearing transplanted MC38 tumors results in CD8^+^ T cell-dependent tumor growth inhibition. Single cell transcriptomics analysis reveals increased proportion of stem-like TCF-1-expressing CD8^+^ T cells among intra-tumoral CD3^+^ cells, a phenotype validated by in vitro lactate treatment of T cells. Mechanistically, lactate inhibits histone deacetylase activity, which results in increased acetylation at H3K27 of the *Tcf7* super enhancer locus, leading to increased *Tcf7* gene expression. CD8^+^ T cells in vitro pre-treated with lactate efficiently inhibit tumor growth upon adoptive transfer to tumor-bearing mice. Our results provide evidence for an intrinsic role of lactate in anti-tumor immunity independent of the pH-dependent effect of lactic acid, and might advance cancer immune therapy.

## Abstract (web summary)

Lactic acid from glycolytic metabolism of cancer cells has been associated with immune suppressive functions. Here authors show that lactate, when depart from the acidic protons, inhibits histone deacetylases in CD8 + T cells, which turns them into potent anti-tumour immune cells.

## Introduction

Extensive efforts have recently been dedicated to the investigation of metabolites and metabolic processes in the regulation of immune functions^1–3^. Various metabolites (e.g., glucose, fatty acid, amino acid) coordinate with immune signaling pathways to control immune cell function and differentiation^4–8^. Metabolic reprogramming is emerging as a new therapeutic principle to augment immune functions and immunotherapy outcomes^9,10^.

Lactate has historically been known as a metabolic waste product from fermentation of carbohydrates or anaerobic glycolysis in skeletal muscles during exercise^11,12^. Over a century ago, Otto Warburg noted cancer cells rapidly produce lactic acid even in the presence of oxygen, a process known as aerobic glycolysis^13,14^. The immune suppressive functions have been reported for lactic acid in glycolytic tumors^15–17^. Recent studies in human lung cancer patients show lactate can be used as an energy source by the cancer cells^18,19^. Further reports demonstrate lactate can overtake glucose as a primary carbon fuel source for a majority of tissues including immune organs^20,21^.

The effect of lactate on CD8^+^ T cell immune functions is not well understood with immune suppressive functions reported for lactic acid in glycolytic tumors^19–21^. In this study, we investigate the effect of sodium lactate, apart from its acidic counterpart, on immune functions. Contrary to the immune suppressive effect of lactic acid, we uncover an immune protective role of sodium lactate through the boosting of stem-like CD8^+^ T cells in cancer treatment. This discovery sheds new light on metabolic reprogramming of immune functions and differentiates the role of lactate and tumor acidity on antitumor immunity.

## Results

### Lactate promotes antitumor immunity through CD8^+^ T cells in multiple tumor models

**Fig. 1:**
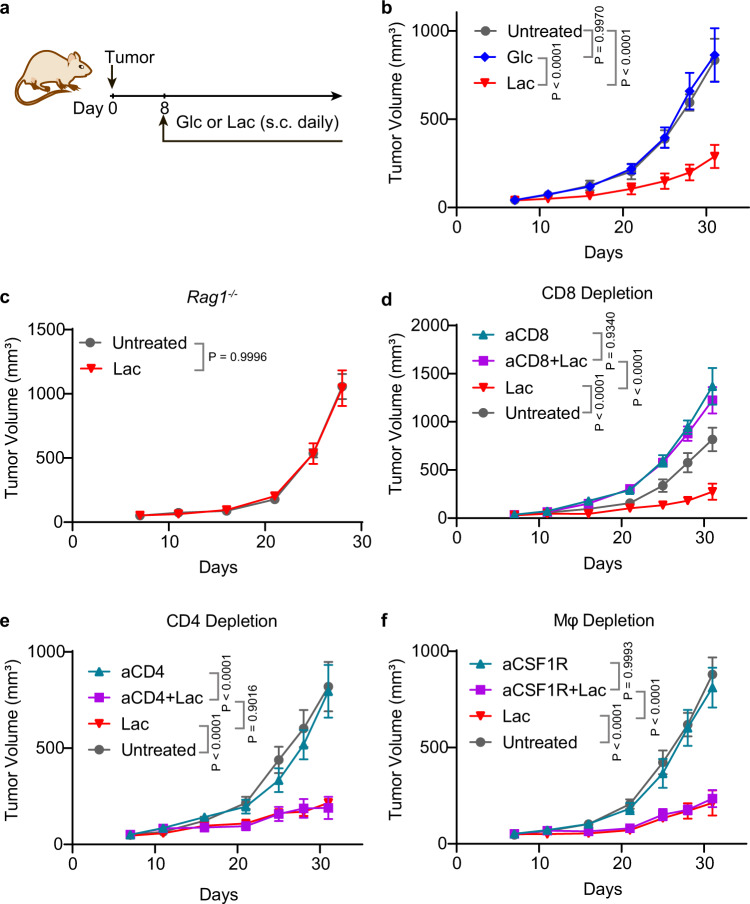
Lactate augments antitumor immunity through CD8^+^ T cells. **a** Treatment regimen for glucose (Glc) or lactate (Lac). Glucose (5 g/kg) or lactate (1.68 g/kg) was subcutaneously administrated daily from Day 8 after tumor inoculation. **b** Tumor growth curve of MC38 tumor model treated with glucose or lactate. C57BL/6 mice (*n* = 6) were inoculated with 1 × 10^6^ MC38 tumor cells and treated with glucose or lactate. **c**, Tumor growth curves of MC38 tumor in B6.129S7-*Rag1*^*tm1Mom*^ (*Rag1*^−/−^) mice treated with lactate. *Rag1*^−/−^ mice (*n* = 7) were inoculated with 1 × 10^6^ MC38 tumor cells and treated with lactate. CD8^+^ T cell (**d**), CD4^+^ T cell (**e**) and macrophage (**f**) depletion assay in MC38 tumor model. C57BL/6 mice (*n* = 6) were inoculated with 1 × 10^6^ MC38 tumor cells and treated with lactate. Anti-CD8 (10 mg/kg) was administered on day 6 and then every three days until the end of the experiment. Anti-CD4 (10 mg/kg) or anti-CSF1R (20 mg/kg) was administered on day 3 and then every three days until the end of the experiment. Data are shown as means ± SEM. P value was determined by one-tail two-way ANOVA with correction using Geisser-Greenhouse method. Source data are provided in Source Data file.

To elucidate the effect of lactate on anti-tumor immune response, we treated tumor bearing mice with subcutaneous (s.c.) administration of sodium lactate solution (1.68 g/kg, pH 7.4, Fig. 1a. All future references to lactate refer to sodium lactate unless otherwise specified). Glucose solution (5 g/kg, pH 7.4) was used as a control. Both injections were prepared as physiologically isotonic solutions. In the MC38 colon cancer model, lactate treatment suppressed tumor growth significantly, while in contrast, glucose had minimal effect (Fig. 1b). The immune cell dependence was tested on three major immune cell populations (CD8^+^ T cells, CD4^+^ T cells and macrophages) in tumor microenvironment. Lactate treatment shows no effect on the tumor growth in *Rag1*^−/−^ mice, indicating that T cells are necessary for the lactate-induced tumor growth inhibition (Fig. 1c). Depletion of CD8^+^ T cells abolished the antitumor effect of lactate (Fig. 1d). In contrast, the anti-tumor efficacy of lactate was not affected by the blockings of CD4^+^ T cells or macrophages (Fig. 1e, f). These results clarify that lactate promotes anti-tumor immunity through CD8^+^ T cells.

**Fig. 2:**
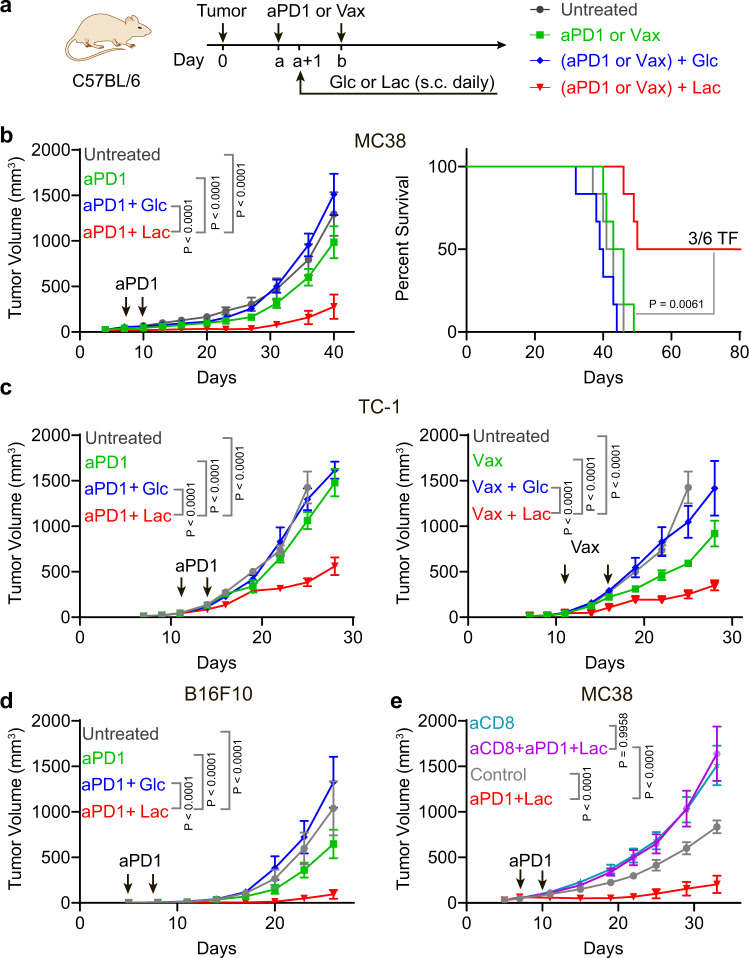
Lactate but not glucose promotes antitumor immunity in multiple tumor models. **a** Treatment regimen for immunotherapy in combination with glucose (Glc) or lactate (Lac). Glucose (5 g/kg) or lactate (1.68 g/kg) was administrated subcutaneously daily one day after the first dose of anti-PD-1 (aPD1, i.p. injection) or PC7A vaccine (Vax, s.c. injection) treatment. **b** Tumor growth and survival data of anti-PD-1 combined with glucose or lactate in MC38 tumor model. C57BL/6 mice (*n* = 6) were inoculated with 1 × 10^6^ MC38 tumor cells and treated with anti-PD-1 (10 mg/kg, day 7 and 10) in combination with glucose or lactate. TF: tumor free. **c** Tumor growth curves of anti-PD-1 or PC7A vaccine combined with lactate or glucose in TC-1 tumor model. C57BL/6 mice (*n* = 6) were inoculated with 1.5 × 10^5^ TC-1 tumor cells and treated with anti-PD-1 (10 mg/kg, day 11 and 14) or PC7A vaccine (0.5 μg E7 peptide, day 11, 16) in combination with glucose or lactate. **d**, Tumor growth curve of anti-PD-1 combined with lactate or glucose in B16F10 tumor model. C57BL/6 mice (*n* = 6) were inoculated with 1.5 × 10^5^ B16F10 tumor cells and treated with anti-PD-1 (10 mg/kg, day 5 and 8) in combination with glucose or lactate. **e** CD8^+^ T cell depletion assay in MC38 tumor model. C57BL/6 mice (*n* = 5) were inoculated with 1 × 10^6^ MC38 tumor cells and treated with anti-PD-1 (10 mg/kg, day 7 and 10) in combination with glucose or lactate. Anti-CD8 (10 mg/kg) was administered at day 6 and then every three days until the end of the experiment. Data are shown as means ± SEM. *P*-value was determined by logrank test (**b**) or one-tail two-way ANOVA with correction using Geisser-Greenhouse method (**a**, **b–e**). ns: not significant. Source data and summary of all P values are provided in Source Data file.

While lactate shows potent tumor growth inhibition in multiple animal cohorts and tumor models, no complete response was achieved with single agent treatment (Fig. 1 and Supplementary Fig. 1 a, b). To further explore a more potent antitumor effect for mechanistic study and potential clinical application, we employed two immunotherapy regimens, which consist of anti-PD-1 as an example of checkpoint blockade therapy or PC7A nanovaccine for T cell therapy^22^ in three murine tumor models. Glucose solution was used as a control (Fig. 2a).

In the MC38 model, lactate treatment significantly improved the efficacy of anti-PD-1 therapy with slower tumor growth and prolonged survival (Fig. 2b). Combination of lactate and anti-PD-1 treatment resulted in tumor-free outcomes in 50% of mice while all mice were lost in other groups before day 60. In contrast, glucose failed to improve the anti-tumor efficacy of anti-PD-1 (Fig. 2b). A delayed initial treatment (from day14) was also tested. Lactate shows similar anti-tumor efficacy and benefits the anti-PD1 therapy (Supplementary Fig. 1c). In the TC-1 tumor model which is transfected with human papilloma virus (HPV) E6/7 proteins, we observed a similar boosting effect of lactate on anti-PD-1 efficacy whereas glucose showed no effect (Fig. 2c). We used E7_43–62_-PC7A nanovaccine (Vax) which primes an E7-specific CD8^+^ T cell response in the TC-1 tumor model^22^. Lactate treatment significantly improved the anti-tumor efficacy of the nanovaccine. In contrast, glucose injection abolished the anti-tumor efficacy of the nanovaccine (Fig. 2c and Supplementary Fig. 1b). In the B16F10 melanoma model, lactate treatment also significantly improved the efficacy of anti-PD-1 while glucose had a slight opposite effect (Fig. 2d). Similar to single agent therapy, the efficacy of combination therapy is dependent on CD8^+^ T cells but not on CD4^+^ T cells or macrophages (Fig. 2e and Supplementary Fig. 1d). Overall, these data suggest that lactate boosts anti-tumor immunity through CD8^+^ T cells in vivo.

### Lactate treatment increases tumor-infiltrating CD8^+^ T cells

**Fig. 3:**
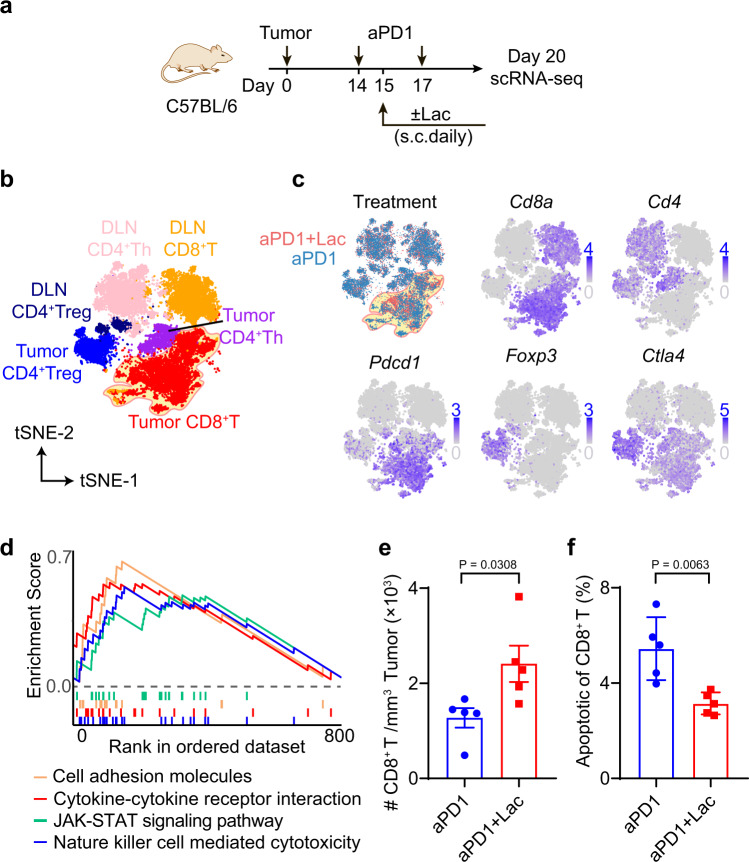
Lactate treatment increases infiltrating CD8^+^ T cells in MC38 tumors. **a** Experimental design of single cell transcriptomic analysis of anti-PD-1 with or without lactate. C57BL/6 mice were inoculated with 1 × 10^6^ MC38 tumor cells and treated with anti-PD-1 (10 mg/kg, day 14 and 17) and lactate (1.68 g/kg, s.c. daily from day 15 to 19). Tumor and tumor draining lymph nodes were harvested on day 20 and analyzed by single cell RNA sequencing using the 10x platform. **b** tSNE plot of T cell clusters with location and cell type information analyzed with Seurat v3.0.1. DLN: tumor draining lymph nodes. **c** Distribution of T cells from different treatments and expression of marker genes. **d** Significantly upregulated pathways in tumor infiltrating CD8^+^ T cells after lactate treatment by unbiased gene set enrichment analysis (gene set database: c2.cp.kegg.v7.2.symbols). **e** Validation of increased tumor infiltrating CD8^+^ T cells by flow cytometry. C57BL/6 mice (*n* = 5) were inoculated with 1 × 10^6^ MC38 tumor cells and treated with anti-PD-1 (10 mg/kg, day 14 and 17) and lactate (1.68 g/kg, s.c. daily from day 15 to 19). MC38 tumors were harvested on day 20 and analyzed by flow cytometry. **f** Analysis of apoptosis markers of CD8^+^ T cells in tumor microenvironment by flow cytometry. Data are shown as means ± SEM. P value was determined by two-tail unpaired *t*-test (**e**, **f**). Source data are provided in Source Data file.

To elucidate the T cell immune microenvironment of lactate-augmented anti-tumor immunity, we analyzed CD3^+^ T cells from MC38 tumors and tumor draining lymph nodes (DLNs) using single cell RNA sequencing analysis (sc-RNAseq, 10x genomics platform) after treatment by anti-PD-1 alone or combined with lactate (Fig. 3a). A delayed initial treatment (from day 14) was used to make sure we collect sufficient tumor tissue for downstream analysis. Overall, 21,826 single CD3^+^ T cells from both treatment conditions were subjected to the following analysis. First, we performed unsupervised graph-based clustering analysis with Seurat^23^ to define major phenotypic T cell populations, which revealed 21 clusters and were visualized by t-distributed stochastic neighbor embedding (tSNE) algorithm (Fig. 3b and Supplementary Fig. 2a). Using well-established marker genes (e.g., *Cd8a, Pdcd1, Cd4, Foxp3, Ctla4*, etc.), we identified 11 CD8^+^ T cell, 7 CD4^+^ helper T cell (CD4^+^ Th), and 3 CD4^+^ regulatory T cell (CD4^+^ Treg) clusters (Fig. 3 b, c and Supplementary Fig. 2, Supplementary Data 1 for cell count in each cluster).

Lactate treatment significantly increased the total number of tumor infiltrating CD8^+^ T cells (Fig. 3c and Supplementary Data 1). Gene set enrichment analysis of tumor infiltrating CD8^+^ T cells showed that lactate treatment induced a significant upregulation of T cell function and signaling related genes (e.g., *Fasl*, *Gzmb*, *Prf1*, *Il2ra*, *Ifngr1*, *Il*7*r* and *Ccl3*) and pathways (e.g., JAK-STAT and cytokine-cytokine receptor interaction, Fig. 3d and Supplementary Fig. 3). Selected genes (*Ccl3, Il7r, Gzmb*) elevated by lactate treatment in the single cell analysis were further verified in OT-I T cells in cell culture by qPCR analysis (Supplementary Fig. 4). Flow cytometry analysis validated the increased CD8^+^ T cell infiltration in MC38 tumors following lactate treatment (Fig. 3e). Furthermore, lactate treatment significantly reduced the percentage of apoptotic CD8^+^ T cells as gated by cleaved Caspase 3 (Fig. 3f).

The 11 CD8^+^ T cell clusters follow a typical differentiation process from naïve to exhausted states observed during anti-tumor immune response (Supplementary Fig. 2, Supplementary Data 1, see Supplementary Data 2 for marker gene list). Among them, we identified 2 naïve T cell clusters (CD8-1 and CD8-2), 2 stem-like (pre-exhausted) T cell clusters (CD8-5 and CD8-6), 4 exhausted T cell clusters (CD8-7 through CD8-10) and 3 cell clusters in intermediate status (CD8-3, CD8-4 and CD8-11). Chi-square analysis (shown as ratio of observed to expected cell numbers, R_O/E_) revealed that lactate treatment led to a consistent increase in cell numbers in stem-like T cell clusters in tumor while exhausted clusters responded differentially (Supplementary Fig. 2b and Supplementary Data 1).

### Lactate treatment increases stem-like CD8^+^ T cell population in MC38 tumors

**Fig. 4:**
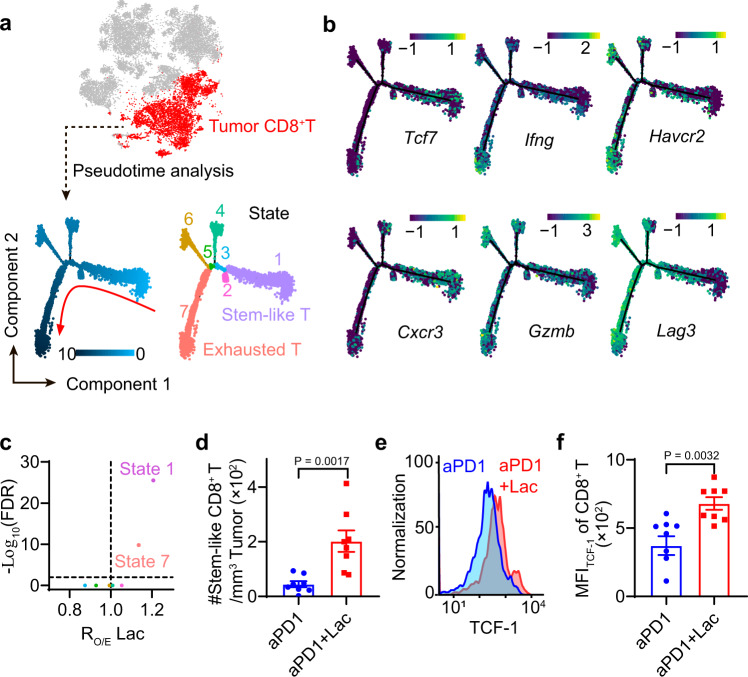
Lactate treatment increases stem-like CD8^+^ T cell population in MC38 tumors. **a** Pseudotime trajectory of CD8^+^ T cells in tumors identifies the differentiation process and distinct states of CD8^+^ T cell subtypes. **b** Labeling of top marker genes on pseudotime trajectory identifies the cells in state 1 are stem-like T cells while those in state 7 are exhausted T cells. **c** Change of CD8^+^ T cell fraction with aPD1+Lac verses aPD1 treatment. R_O/E_ Lac analysis showed lactate treatment increased the T cell populations in states 1 and 7. **d** Validation of increase in TCF1^+^ PD1^+^ CD8^+^ T cell population after lactate treatment by flow cytometry. C57BL/6 mice (*n* = 8) were inoculated with 1 × 10^6^ MC38 tumor cells and treated with anti-PD-1 (10 mg/kg, day 14 and 17) and lactate (1.68 g/kg, s.c. daily from day 15 to 19). Tumors were harvested on day 20 and analyzed by flow cytometry. **e**, **f** Flow cytometry plot and quantification of TCF-1 expressions in tumor-infiltrating CD8^+^ T cells after different treatments. Samples are the same as described in D. Data are shown as means ± SEM. P value was determined by one-tail Chi-square test (**c**) or two-tail unpaired *t*-test (**d**, **f**). Source data are provided in Source Data file.

To further investigate the effect of lactate on different CD8^+^ T cell subtypes, we analyzed the differentiation trajectory of tumor infiltrating CD8^+^ T cells using pseudotime analysis by Monocle 2 (Fig. 4a and Supplementary Fig. 5a)^24^. The trajectory was divided into 7 states with unsupervised clustering, which begins with a stem-like state 1 and differentiates into exhausted states. Stem-like CD8^+^ T cells in state 1 have high abundance of stem-like genes (*Tcf7*, *Il7r*, *Cxcr3*) and low abundance of effector genes (*Ifng*, *Gzmb*, *Gzmc*, *Gzmf*) and exhaustion genes (*Lag3*, *Pdcd1*, *Havcr2*, *Entpd1*) (Fig. 4b, Supplementary Fig. 5b, c and Supplementary Data 3).

We observed that lactate treatment resulted in the most significant increase in the number of stem-like CD8^+^ T cells (state 1) over all other T cell states (Fig. 4c). We used flow cytometry to confirm the increase in the number of tumor infiltrating stem-like T cells (Fig. 4d) and elevated expression of the TCF-1 protein in the total population of tumor infiltrating CD8^+^ T cells (Fig. 4 e, f, histograms and controls are shown in Supplementary Fig. 6a) in response to lactate treatment. Collectively, these results show that lactate treatment increased the number of stem-like T cells and elevated the expression of *Tcf7*/TCF-1 in CD8^+^ T cells in vivo.

### Lactate increases TCF-1 expression and reduces apoptosis of CD8^+^ T cells during ex vivo expansion

**Fig. 5:**
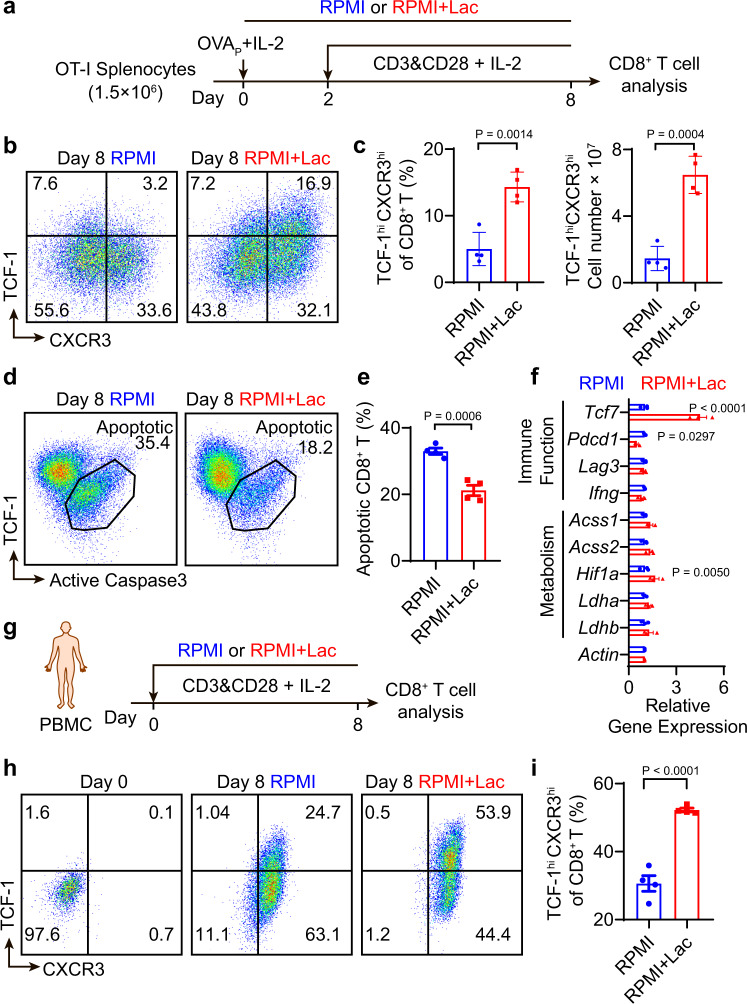
Lactate increases TCF-1 expression and reduces apoptosis of CD8^+^ T cells during ex vivo expansion. **a** Experimental design of ex vivo OT-I CD8^+^ T cell expansion. Fresh splenocytes from C57BL/6-Tg(TcraTcrb)1100Mjb/J mice were primed with SIINFEKL peptide (1 μg/mL) and hIL-2 (50 U/mL) for two days and stimulated with anti-CD3 and anti-CD28 (0.5 μg/mL each) from day 3 to 8 with hIL-2 (30 U/mL). **b**, **c** Flow cytometry plots and quantification of TCF-1^hi^CXCR3^hi^ population of OT-I CD8^+^ T cells on day 8 of ex vivo expansion (*n* = 4 biologically independent samples). **d**, **e** Quantification of percentage of apoptotic OT-I CD8^+^ T cells by flow cytometry on day 8 of ex vivo expansion (*n* = 4 biologically independent samples). **f**, Relative gene expression in OT-I CD8^+^ T cells on day 4 detected by RT-PCR (n = 3 biologically independent samples). **g** Experimental design of ex vivo expansion of CD8^+^ T cells from human PBMCs. PBMCs from cord blood were activated and cultured in the presence of anti-CD3 and anti-CD28 beads (T cell: Beads = 1: 1) supplemented with hIL-2 (30 U/mL). **h**, **i** Flow cytometry plots and quantification of TCF-1^hi^CXCR3^hi^ population of human CD8^+^ T cells on day 8 of ex vivo expansion (*n* = 4 biologically independent samples). Data are shown as means ± SEM. *P*-value was determined by two-tail unpaired *t*-test (**c**, **e**, **i**) or one-tail two-way ANOVA without correction (**f**). Source data are provided in Source Data file.

To elucidate the effect of lactate on CD8^+^ T cells, we first investigated whether lactate impacts the ex vivo culture of CD8^+^ T cells derived from mouse splenocytes or human peripheral blood mononuclear cells (PBMCs). OT-I splenocytes were obtained from C57BL/6-Tg(TcraTcrb)1100Mjb/J (OT-I transgenic) mice and stimulated with OVA peptide (OVA_p_, SIINFEKL) and IL-2 for two days, followed by cell culture with anti-CD3, anti-CD28 and IL-2 (Fig. 5a). The dose of lactate for in vitro T cell culture was set to 40 mM to mimic the lactate concentration in tumor interstitial fluid (TIF) (Supplementary Fig. 6b). Lactate treatment significantly increased the number of TCF-1^hi^CXCR3^hi^ cells and mean fluorescent intensity of TCF-1 in CD8^+^ T cells compared to control condition (RPMI medium containing 2 mM lactate, Fig. 5 b, c and Supplementary 6c). Increased TCF-1 expression by lactate was also observed in memory T cells cultured with IL-15 (Supplementary 6d)^25^. In CD44^hi^ CD62L^hi^ central memory T cells, sodium lactate treatment led to a 26% increase of TCF-1 expression on day 8 of the culture. Similarly, in IL-2 induced effector T cells, sodium lactate treatment leads to a 30% increase of TCF-1 expression on day 8 of the culture. Comparable to our in vivo observation (Fig. 3f), lactate treatment during ex vivo expansion reduced the percentage of apoptotic CD8^+^ T cells (Fig. 5d, e). Of note, by examining the co-staining of TCF-1 and active caspase 3, apoptotic CD8^+^ T cells were enriched in the low TCF-1 expressing population (Fig. 5d). This result is consistent with reports of *Bcl2* induction by *Tcf7* upregulation, which inhibits apoptosis^26–28^. Finally, we quantified the mRNA expressions for genes of interest during the T cell expansion by real-time polymerase chain reaction (PCR). Lactate treatment significantly increased the expression level of *Tcf7* mRNA, while insignificant differences were observed with activation/exhaustion related genes including *Pdcd1*, *Lag3* and *Ifng* and metabolism related genes including *Acss1*, *Acss2*, *Hif1a*, *Ldha and Ldhb* (Fig. 5f).

To study the lactate effect on human CD8^+^ T cells, we employed PBMCs isolated from human cord blood. PBMCs were cultured in lactate-augmented (40 mM) or control RPMI medium with anti-CD3 and anti-CD28 modified microbeads and IL-2 for 8 days (Fig. 5g). Similar to the results from OT-I CD8^+^ T cells, lactate treatment significantly increased the TCF-1^hi^CXCR3^hi^ cell population and the mean florescent intensity of TCF-1 protein in human CD8^+^ T cells (Fig. 5h, i and Supplementary Fig. 6c). In contrast, lactic acid, the conjugated acid form of lactate, resulted in significant CD8^+^ T cells death in vitro at 40 mM in RPMI medium (pH = 4.6) and reduced the induction effect of lactate on TCF-1 expression at pH 6.9 (Supplementary Fig. 6e, f).

### Lactate induces T cell stemness through epigenetic regulation

**Fig. 6:**
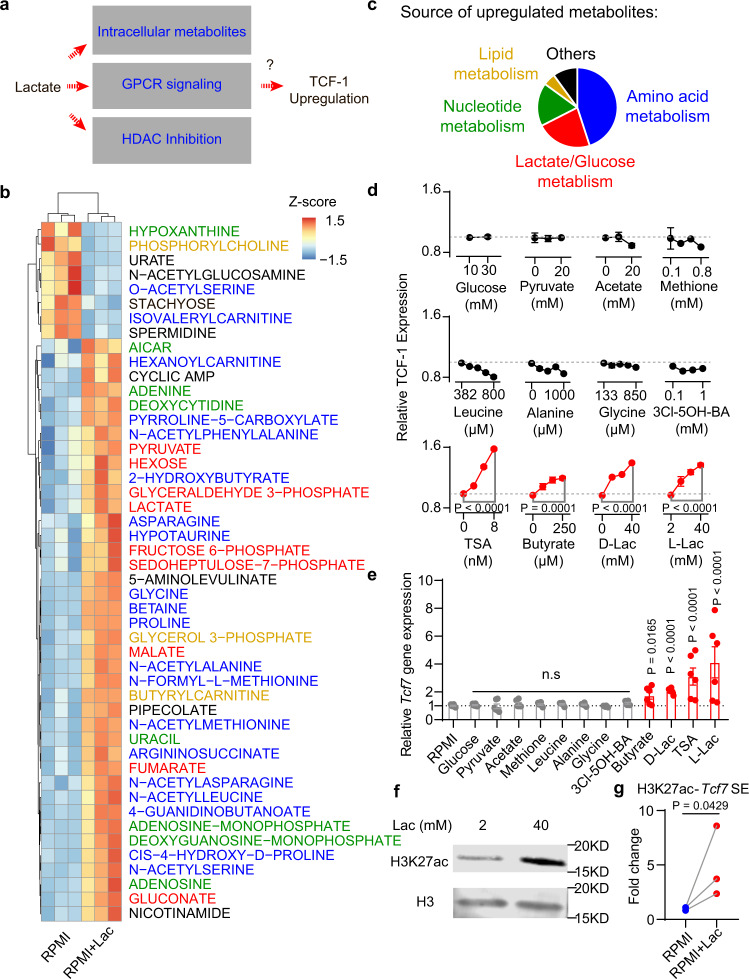
Lactate increases TCF-1 expression through inhibition of histone deacetylases (HDAC). **a** Potential mechanisms for lactate induced TCF-1 upregulation. **b** Heatmap of significantly changed metabolites in CD8^+^ T cells treated with or without 40 mM lactate. **c** Source of upregulated metabolites with 40 mM lactate treatment. **d** TCF-1 protein expression in CD8^+^ T cells treated by different metabolites, GPCR agonist or HDAC inhibitors (*n* = 3 biologically independent samples). **e** Gene expression of *Tcf7* in CD8^+^ T cells treated by different metabolites, GPCR agonist or HDAC inhibitors. The concentration of different metabolites was the same as the highest concentration in **d** (*n* = 6 biologically independent samples). **f** Western blot of histone H3K27ac of CD8^+^ T cells cultured with or without 40 mM lactate (One representative data was shown from 3 independently repeated experiments). **g** Quantification of H3K27ac enrichment by CUT&RUN PCR at the *Tcf7* super enhancer locus (*n* = 3 biologically independent samples). Data are shown as means ± SEM. *P*-value was determined by two-tail unpaired *t*-test without adjustment (**d**, **e**) or two-tail ratio paired *t*-test (**g**). Source data are provided in Source Data file.

Besides its newly uncovered role as a primary carbon fuel source, lactate has additional functions as an agonist to G-protein-coupled receptor (GPCR) signaling and as an inhibitor to histone deacetylase (HDAC). As a carbon fuel source, lactate is converted to pyruvate by lactate dehydrogenase B and further fuels the TCA cycle^19,20^. As a GPCR agonist (e.g., GPR81), lactate regulates multiple biological processes through the secondary messenger cyclic AMP (cAMP)^29,30^. Also, lactate has been reported as an HDAC inhibitor with an IC_50_ of 40 mM^31^. The increase of TCF-1 expression in CD8^+^ T cells may arise from lactate augmented metabolites (e.g., pyruvate, amino acids), lactate-mediated GPCR signaling or HDAC inhibition (Fig. 6a).

To investigate the biochemical mechanism, we first measured the abundance of ∼200 common metabolites in CD8^+^ T cells (OT-I) cultured with lactate-augmented (40 mM) or control RPMI medium (Fig. 6b). Lactate treatment boosted central carbon metabolism, including a variety of metabolites in glucose/lactate, amino acid, nucleotide and lipid metabolisms (Fig. 6b, c and Supplementary Data 4). The increased abundance of lactate, pyruvate, TCA cycle intermediates and multiple amino acids correlates with lactate influx, conversion to pyruvate and subsequent utilization in the TCA cycle as shown by the stable isotope tracing study (Supplementary Fig. 7 and Supplementary Data 5). In regards to the GPCR signaling pathway, lactate treatment increased cAMP levels in the T cells.

We next examined the expression of TCF-1 in the CD8^+^ T cells as a function of selected metabolites or a GPR81 agonist (Fig. 6d, e). Increasing glucose concentration from 10 to 25 mM in RPMI medium showed no effect on TCF-1 expression. Pyruvate (0-20 mM), the direct metabolite downstream of lactate conversion in cells, did not cause increase of TCF-1. Acetate, another monocarboxylate species that can be synthesized from pyruvate and fuel TCA cycle through acetyl-CoA, also had little effect. Lactate augmented amino acids, including essential amino acids (e.g., Methionine and Leucine) and nonessential amino acids (e.g., Glycine and Alanine), also did not increase TCF-1 expression. A GPR81 ligand, 3-chloro-5-hydroxybenzoic acid (3Cl-5OH-BA), which stimulates cAMP production similar to lactate-induced effect^30,32^, didn’t increase TCF-1. These results indicate lactate mediated HDAC inhibition as a potential driver for increased T cell stemness.

Lactate has been reported as an endogenous chemical inhibitor (IC_50_ = 40 mM) of HDAC that links the metabolic state of cells to gene transcription^31^. To further corroborate the role of HDAC inhibition on T cell stemness, OT-I CD8^+^ T cells were treated with known HDAC inhibitors including trichostatin A (TSA), D-lactate (all other lactates refer to L-lactate unless noted otherwise), or butyrate. Flow cytometry and qPCR analyses showed elevated TCF-1 expressions similar to the treatment outcome by 40 mM lactate (Fig. 6d, e). Epigenomic profiling identified acetylation of H3K27 at the *Tcf7* super enhancer site correlates with the naïve and central memory identity of CD8^+^ T cells^33^. Western blot of lactate treated CD8^+^ T cells showed 3.5-fold increase of acetylation at the H3K27 site (Fig. 6f). Cleavage under targets and release using nuclease (CUT&RUN) PCR analysis further confirmed significant H3K27ac enrichment at the *Tcf7* super enhance locus in the lactate treated CD8^+^ T cells (Fig. 6g).

Collectively, these results indicate that lactate directly induces CD8^+^ T cell stemness through epigenetic regulation of cell identity.

### Adoptive transfer of lactate-pretreated CD8^+^ T cells achieves potent tumor growth inhibition in vivo

**Fig. 7:**
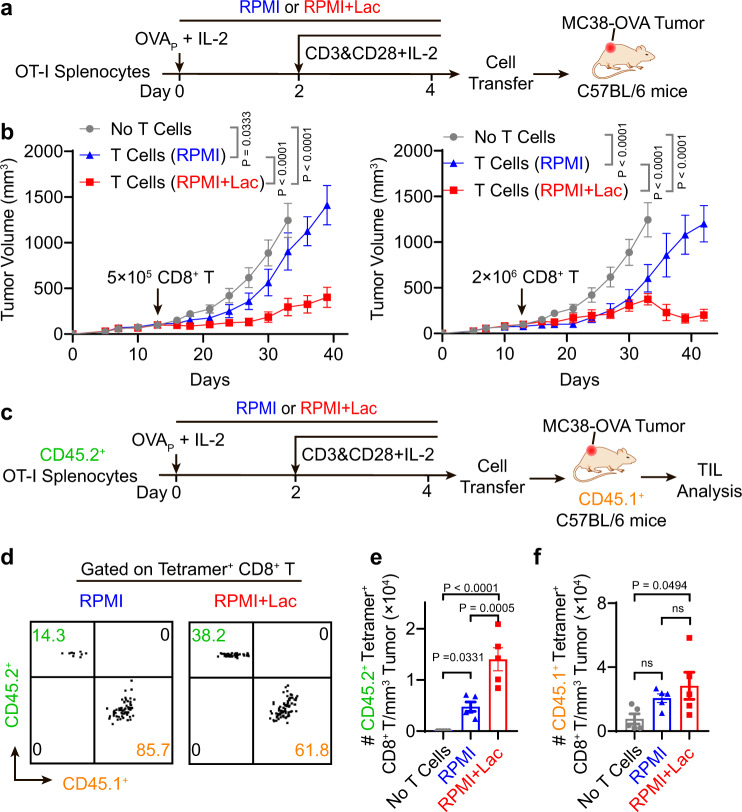
CD8^+^ T cells expanded under high lactate condition show potent tumor growth inhibition in vivo. **a** Treatment regimen of T cell receptor engineered T cell therapy (TCR-T). OT-I splenocytes were cultured ex vivo for 4 days with or without 40 mM sodium lactate in the culture medium. After purification with negative selection magnetic beads, CD8^+^ T cells were transferred to MC38-OVA tumor bearing mice. Average tumor size is above 100 mm^3^ at the time of cell transfer. **b** The growth curves of MC38-OVA tumor model were significantly inhibited after transfer of TCR-T (5 × 10^5^ or 2 × 10^6^) pretreated with sodium lactate (*n* = 5). **c** Analysis of tumor-infiltrating T cells after adoptive TCR-T cell transfer. Seven days after the cell transfer, tumor-infiltrating lymphocytes were analyzed by flow cytometry. **d**–**f** Flow cytometry plot and quantification of transferred (CD45.2^+^) and endogenous (CD45.1^+^) OVA-tetramer^+^ CD8^+^ T cells show lactate pretreatment significantly increased the number of transferred CD8^+^ T cells (CD45.2^+^) but not endogenous CD8^+^ T cells (CD45.1^+^) in the MC38-OVA tumors (*n* = 5). Data are shown as means ± SEM. *P*-value was determined by one-tail two-way ANOVA with correction using Tukey method (**b**, **c**) or one-tail one-way ANOVA with correction using Geisser-Greenhouse method (**e**, **f**). Source data are provided in Source Data file.

Recent studies have linked TCF-1 expressing stem-like CD8^+^ T cells, which retain polyfunctionality and persistence of cytotoxic function, as the key T cell subsets which respond to adoptive cell transfer therapy, anti-PD-1 and vaccination therapy^34–40^. To investigate whether lactate-induced TCF-1 expressing CD8^+^ T cells can improve tumor therapy, we harvested and intravenously injected OVA-specific CD8^+^ T cells into MC38-OVA tumor bearing mice (Fig. 7a). OVA-specific T cells pretreated with lactate ex vivo demonstrated significantly improved tumor growth inhibition over those cultured in control RPMI medium (Fig. 7b). To further investigate the effect of transferred vs. endogenous OVA-specific CD8^+^ T cells on antitumor efficacy, we employed OVA tetramer^+^ CD8^+^ T cells from CD45.2 expressing donors (OT-I mice) and transferred them to CD45.1 expressing tumor bearing mice (Fig. 7c). Seven days after cell transfer, we analyzed the number of host (CD45.1^+^) and donor (CD45.2^+^) CD8^+^ T cells in tumors by flow cytometry. Lactate pretreatment significantly expanded CD45.2^+^ tetramer^+^ CD8^+^ T cells to a larger clonal size over those from control RPMI medium (Fig. 7 d, e). In contrast, the number of endogenous CD45.1^+^ tetramer^+^ CD8^+^ T cells showed no significant difference after transfer of CD45.2^+^ T cells with and without lactate pretreatment (Fig. 7d, f).

These data support that lactate-pretreated CD8^+^ T cells, which have high TCF-1 expression, show improved anti-tumor immune response in vivo.

## Discussion

In this study, we report a mechanistic link between lactate, CD8^+^ T cell stemness and improved outcome of cancer immunotherapy. Recent studies show lactate overtakes glucose as the primary fuel source of most tissues^20,21^. The effect of lactate on antitumor immunity is not well understood with immune suppressive functions reported for lactic acid^15–17^. Our side-by-side comparison between subcutaneous administration of high dose sodium lactate versus glucose revealed an immune protective role of lactate (Figs. 1 and 2). We theorize the underappreciated immune protective role of lactate is caused by the dominant immunosuppressive effect of tumor acidity. In the highly glycolytic and poorly vascularized microenvironment of solid tumors, glucose is preferentially converted to lactic acids by the cancer cells, which are then secreted to the extracellular environment through monocarboxylate transporters^41–43^. The accumulating lactic acids lead to elevated concentrations of lactate and tumor acidity. Using an in vitro T cell culture model, we and others show significant CD8^+^ T cell death induced by 40 mM lactic acid in the RPMI medium (pH = 4.6) (Supplementary Fig. 6e) or hydrochloric acid (pH = 5.8)^16^, while 40 mM sodium lactate in the RPMI medium (pH = 7.4) protected CD8^+^ T cell viability (Fig. 5d, e). At mildly acidic pH (6.9), addition of 40 mM lactate while maintaining medium pH increased TCF-1 expressions in CD8^+^ T cells. However, lowering the medium pH from 7.4 to 6.9 was sufficient to abolish the lactate-induced increase of T cell stemness observed at pH 7.4 (Supplementary Fig. 6f), further corroborating that environmental acidity can override lactate effect in the elevation of T cell stemness. In in vivo animal studies, sodium lactate treatment increased the lactate dose without elevating tumor acidity, which led to the increased stemness of CD8^+^ T cells and tumor growth inhibition (Figs. 1 and 2). Lactate-mediated antitumor efficacy is largely driven by the CD8^+^ T cells in the MC38 tumors as shown by the abrogated effect in mice with CD8^+^ T cell depletion (Fig. 1d).

Lactate has broad physiological functions in carbon metabolism, cell signaling and epigenetic regulation^20,29–31,44^. Our mechanistic studies reveal lactate-mediated HDAC inhibition is primarily responsible for increased stemness of CD8^+^ T cells. In contrast, glucose, lactate-augmented metabolites (e.g., pyruvate, various amino acids) or GPCR signaling (e.g., GPR81 agonist) did not appear to contribute to the T cell stemness. Known HDAC inhibitors (e.g., TSA, butyrate) also increased T cell stemness in the current experimental conditions (Fig. 6d, e). However, prolonged exposure at elevated concentrations of these agents resulted in decreased viability of T cells (Supplementary Fig. 8). In contrast, ex vivo expansion of CD8^+^ T cells under high lactate concentrations reduced cell apoptosis over prolonged culture (Fig. 5). In vivo administration of high dose sodium lactate further demonstrates the safety of lactate therapy as indicated by minimal changes of blood pH and body weight (Supplementary Fig. 9).

As a common metabolite, lactate can be utilized by many cell types and will have pleiotropic effects in a context dependent manner. Brown *et al* reported that tumor cell-derived lactatic acid activates GPR81 in dendritic cells and prevents presentation of tumor-specific antigens to other immune cells^45^. Raychaudhuri et al. found intratumoral lactate attenuates type I interferon production in plasmacytoid dendritic cells leading to a tolerogenic phenotype^46^. Feng et al. reported tumor cell-derived lacate induces upregulation of immune checkpoint PD-L1 in human lung cancer cells^47^. Previous studies also show that tumor-derived lactic acid fuels regulatory T cells and induces M2 polarization of macrophages^17,48,49^. Many of these studies relied on tumor-derived lactic acid or knockout models of lactate dehydrogenase A or monocarboxylate transporter, which include the convoluted effects from lactate, tumor acidity and altered cancer cell metabolism. It is conceivable that lactate may also exert opposite effect on these cell populations compared to lactic acid. The delineation of the lactate versus lactic acid effects on other immune cell types is worthy of further investigations.

Endogenous lactate is largely produced during intense exercise. Consistent with our discovery, Johnson *et al* reported that lactate is a potential mediator for exercise-induced immune protection^50^. In the clinic, lactate is widely used as Ringer’s lactate or Hartmann’s solution with well-documented safety profile for fluid resuscitation and for reducing metabolic acidosis. The current Ringer’s formulation or a modified solution with increased lactate concentration may protect CD8^+^ T cell functions during immunotherapy in cancer patients. As a more direct implementation of lactate into the clinical workflow, the inclusion of lactate in ex vivo T cell expansion may benefit chimeric antigen receptor T cell (CAR-T) therapy.

In summary, results from this study revealed the unusual immune protective role of lactate in antitumor immunity. Single cell transcriptomics and flow cytometry analysis show an increased subpopulation of stem-like TCF-1-expressing CD8^+^ T cells upon lactate treatment, which is further confirmed by ex vivo culture of CD8^+^ T cells from mouse splenocytes and human PBMCs. Inhibition of histone deacetylase activity by lactate is responsible for the increased TCF-1 expression on the CD8^+^ T cells. Lactate has the potential to augment the therapeutic outcomes of immune checkpoint blockade, T cell vaccine and adoptive T cell transfer therapy.

## Methods

### Reagents

The details of reagents used are provide in Supplementary Data 6.

### Mice

Male and female (6–10 week) C57BL/6 J(Strain #:000664), NOD.Cg-Prkdc^scid^/J(Strain #:001303), C57BL/6-Tg(TcraTcrb)1100Mjb/J (Strain #:003831), B6.129S7-*Rag1*^*tm1Mom*^/J (Strain #:002216), B6.SJL-Ptprc^a^Pepc^b^/BoyJ (Strain #:002014) mice were purchased from Jackson Laboratory. All mice were maintained in specific pathogen free animal facility with controlled temperature (68–79 ^o^F), humidity (30–70%), and light/dark cycle (lights between 6 am and 6 pm). Mice were euthanized at the end of the experiment with CO_2_ asphyxiation with cervical dislocation as a physical secondary assurance method. All animal experiments were performed with ethical compliance and approval from Institutional Animal Care and Use Committee of the University of Texas Southwestern Medical Center.

### Cells

Murine melanoma B16F10 and colon adenocarcinoma MC38 cell lines were purchased from American Type Culture Collection (ATCC). TC-1 cells were kindly provided by Dr. T. C. Wu at John Hopkins University. MC38-OVA cells were made by lentiviral transduction of OVA gene. All cancer cell lines were routinely tested using e-Myco mycoplasma PCR Detection kit (Bulldog Bio Inc) and cultured in high glucose Dulbecco’s modified Eagle’s medium supplemented with 10% fetal bovine serum, 100 U/mL penicillin, 100 U/mL streptomycin and 1×GlutaMax under 5% CO_2_ at 37 ^o^C. Immune cells from mouse or human were cultured in RPMI 1640 medium supplied with 10% heat-inactivated fetal bovine serum, 100 U/mL penicillin, 100 μg/mL streptomycin, 20 mM HEPES, 50 μM 2-Mercaptoethanol and 1 × GlutaMax.

### Human peripheral blood mononuclear cells

Human cord blood samples were obtained from University of Texas Southwestern (UTSW) Parkland Hospital according to the regulation and the use approval of human cord blood in compliance with UTSW Human Investigation Committe protocol (STU 112010-047). The procedure is approved through a protocol exempt from informed consent as approved by the Institutional Review Board of UTSW and the Office for Human Research Protections (OHRP) supported by the U.S. Department of Health and Human Services. Human peripheral blood mononuclear cells (PBMC) were purified from cord blood by Ficoll-Paque Plus according to the manufacturer’s manual.

### Tumor growth and treatment

C57BL/6 J mice were inoculated with 1 × 10^6^ MC38 tumor cells, or 1.5 × 10^5^ TC-1 tumor cells or 1.5 × 10^5^ B16F10 tumor cells on the right flank on day 0. Same volume (2 mL) of isotonic sodium lactate (150 mM, pH 7.4) or glucose solution (278 mM, pH 7.4) was administered subcutaneously under the dorsal skin near the neck. For MC38 model, animals were intraperitoneally (i.p.) treated with anti-PD-1 (10 mg/kg, day 7 and 10) in combination with glucose or sodium lactate (s.c., 5 g/kg or 1.68 g/kg, respectively) daily, beginning on day 8. For TC-1 tumor model, animals were treated with anti-PD-1 (i.p. 10 mg/kg, day 11 and 14) or PC7A vaccine (s.c. 0.5 μg E7 peptide, day 11 and 16) in combination with glucose or sodium lactate (s.c., 5 g/kg or 1.68 g/kg, respectively) daily, beginning on day 12. For B16F10 tumor model, animals were treated with anti-PD-1 (i.p. 10 mg/kg, day 5 and 8) in combination with glucose or sodium lactate (s.c., 5 g/kg or 1.68 g/kg, respectively) daily, beginning on day 6. For immune cell depletion assay, anti-CD8 antibodies, anti-CD4 antibodies or anti-NK1.1 were administrated every three days during the treatment (i.p. 10 mg/kg). For single cell analysis and in vivo flow cytometry analysis in MC38 tumor model, animals were intraperitoneally (i.p.) treated with anti-PD-1 (10 mg/kg, day 14 and 17) in combination with or without sodium lactate (s.c., 1.68 g/kg) daily, beginning on day 15. Tumor and tumor draining lymph nodes were collected on day 20 for analysis. Tumor volumes were measured with a caliper by the length (L), width (W) and height (H) and calculated as tumor volume = L×W×H/2. Animals were considered dead when tumor volume reached > 1500 mm^3^. Tumor free C57BL/6 J mice were used for body weight study in Supplementary Fig. 7b.

### Lactate concentration in tumor interstitial fluid

Tumor interstitial fluid was collected from freshly resected MC38 tumor. Specimens were centrifuged against a 70 μm cell strainer at 4 °C for 5 min at 300 g. Flow-through tissue interstitial fluid was centrifuged at 4 °C for 5 min at 500 g. Supernatant were flash-frozen and stored at −80 °C before batch analysis. The lactate concentration was determined with lactate assay kit (Sigma-Aldrich, MAK064) according to the manufacturer’s protocol.

### Lactate Concentration in plasma

Mouse blood (1 mL) was collected from lactate treated mice at different time points (0, 0.1, 0.5, 2, 10, and 24 h after lactate injection) using K3 EDTA blood collection tubes. Cells were removed from plasma by centrifugation for 10 mins at 2000 g at 4 ^o^C. Lactate concentration in plasma was measured by BioProfile® FLEX2 (Nova Biomedical). Tumor free C57BL/6 J mice were used for this set of experiment.

### pH in blood

Mouse blood (1 mL) was collected from lactate treated mice at different time points (0, 0.1, 2, 10, and 24 h after lactate injection) to 1.5 mL Eppendorf tubes. The pH was measured immediately after collection with pH meter (Mettler Toledo AG). Tumor free C57BL/6 J mice were used for this set of experiment.

### Single cell RNA sequencing

For single cell RNAseq analysis, we treated 10 mice under each treatment condition (i.e., anti-PD-1 or anti-PD-1+Lactate). We isolated the tumor and a single draining lymph node from each mouse, then pooled cells from each tissue type and proceeded with library preparation. Four pooled libraries (tumor T cells from anti-PD-1 treatment; tumor T cells from anti-PD-1+Lactate treatment; lymph node cells from anti-PD-1 treatment; lymph node cells from anti-PD-1+Lactate treatment, Data_file_S1) were sequenced and analyzed. Tumor-infiltrating CD3^+^ T cells were obtained by microbeads enrichment and flow sorting. Briefly, CD45^+^ cells from tumors were isolated with Tumor Dissociation Kit (Miltenyi) and purified with Dead Cell Removal Kit (Miltenyi) and CD45 (TIL) MicroBeads (Miltenyi) according to the manufacturer’s instructions. The purified cells were further sorted for live CD45^+^CD3^+^ population on BD Aria II SORP sorter. Tumor draining lymph node cells were flow sorted for live CD45^+^ population. Isolated cells from each tissue type and each treatment condition were pooled and proceeded with library preparation individually. Cells were barcoded with Chromium Single Cell 5’ Library & Gel Bead Kit (10x Genomics). Library construction was performed following the protocol of Chromium Single Cell 5’ Library Construction Kit. Libraries were pooled with Chromium i7 Multiplex Kit. The final pooled libraries were sequenced on NovaSeq S4 platform (Novogene).

### Analysis of single cell RNA sequencing data

Sequencing reads were aligned to mouse genome (mm10) and unique molecular identifiers (UMIs) were counted for each gene using Cell Ranger 3.0.1 (10x Genomics) The R software (3.6.1) package Seurat (version 3.0.1) was used for further analysis. Cells with a high or low proportion of detected genes (>2 × median absolute deviations) and those with a high proportion of mitochondrial genes (>15%) were removed. Cells were divided into clusters with graph-based clustering using the Seurat function Find Clusters at resolution 0.8. Marker genes for each cell cluster were calculated with Wilcoxon Rank Sum test with log10 (fold change) > 0.1. Monocle 2 (version 2.10.1) was used to estimate a pseudo-temporal path of tumor infiltrating CD8^+^ T cell differentiation. Monocle object was formed by Monocle implemented newCellDataSet function with lowerDetectionLimit = 0.5. Differentially expressed genes (DEG) were identified by Limma (3.40.5) R package with an absolute log2 fold change (FC) > 0.05 and *q* < 0.01^51^. Pathway enrichment was performed on ranked DEG lists with GSEA using KEGG under gene size 15 and FDR 0.05.

### Ex vivo culture of activated CD8^+^ T cells

For mouse studies, single cell suspensions from lymph nodes and spleens of C57BL/6-Tg (TcraTcrb)1100Mjb/J transgenic mice were activated with SIINFEKL peptide (1 μg/mL) and hIL-2 (50 IU/mL). 48 h post activation, CD8^+^ T cells were further cultured with anti-CD3 and anti-CD28 antibodies. For human studies, human PBMCs were activated and cultured with Dynabeads™ Human T-Activator CD3/CD28 according to manufacturer’s instruction. T cells were passaged every 48 h.

### Flow cytometry analysis

Single cell suspensions were obtained from cell culture or mouse tissues. Mouse tumors were dissociated by Collagenase (1 mg/mL) and DNAse I (0.2 mg/mL) and lymph nodes were dissociated with 70 μm cell strainer. For each single cell suspension, Fc receptor was blocked with anti-FcγIII/II (clone 2.4G2) for 20 min, followed by staining with selective antibodies of cell surface markers and live/dead dyes. Intracellular markers including active caspase 3 and TCF-1 were stained after cell permeabilization with True-Nuclear transcription factor buffer set (BioLegend). Data were collected on BD LSR Fortessa or Beckman CytoFLEX flow cytometer and analyzed by FlowJo (Tree Star Inc., Ashland, OR) software. Gating strategies were listed in Supplementary Fig. 10.

### RNA extraction and quantitative real-time PCR analysis

Total RNA from T cells was extracted with RNeasy Plus Mini Kit and reversed transcribed with iScript-gDNA Clear cDNA Synthesis Kit (Bio-Rad). Real-time PCR was performed with QuantiFast SYBR Green PCR Kit (Qiagen) according to the manufacturer’s instructions and different primer sets on CFX Connect Real-Time PCR Detection System (Bio-Rad). The levels of gene expression were determined with delta-delta Ct method using β-actin as the internal control.

### Metabolomics analysis

Metabolites were extracted from cultured CD8^+^ T cells with 80/20 methanol/water after 3 cycles of freeze-thaw. Data acquisition was performed by reverse-phase chromatography on a 1290 UHPLC liquid chromatography (LC) system interfaced to a high-resolution mass spectrometry (HRMS) 6550 iFunnel Q-TOF mass spectrometer (MS) (Agilent Technologies, CA). Analytes were separated on an Acquity UPLC® HSS T3 column (1.8 μm, 2.1 × 150 mm, Waters, MA) at room temperature. Mobile phase A composition was 0.1% formic acid in water and mobile phase B composition was 0.1% formic acid in 100% acetonitrile. The LC gradient was 0 min: 1% B; 5 min: 5% B; 15 min: 99% B; 23 min: 99% B; 24 min: 1% B; 25 min: 1% B. The flow rate was 250 μL/min. The sample injection volume was 5 μL. The MS was operated in both positive and negative (ESI + and ESI-) modes. ESI source conditions were set as follows: dry gas temperature 225 °C and flow 18 L/min, fragmentor voltage 175 V, sheath gas temperature 350 °C and flow 12 L/min, nozzle voltage 500 V, and capillary voltage +3500 V in positive mode and −3500 V in negative. The instrument was set to acquire over the full m/z range of 40–1700 in both modes, with the MS acquisition rate of 1 spectrum/s in profile format. Raw data were processed using Profinder B.08.00 SP3 software (Agilent Technologies, CA) with an in-house database containing retention time and accurate mass information on 600 standards from Mass Spectrometry Metabolite Library (IROA Technologies, MA) which was created under the same analysis conditions. The in-house database matching parameters were: mass tolerance 10 ppm; retention time tolerance 0.5 min. Peak integration result was manually curated in Profinder for improved consistency and exported as a spreadsheet. Data were filtered and analyzed with Metaboanalyst 5.0 and plotted with R 4.0.1. Cut-off for significant change was set to FDR 0.1, fold change 1.5.

### Stable isotope labeling

Activated CD8^+^ T cells from OT-1 mice were rinsed in PBS, then cultured with medium containing the isotopically labeled glucose or lactate for 8 h. For analysis of intracellular metabolites by GC-MS, 2 × 10^6^ cells were rinsed in ice-cold normal saline and lysed with three freeze-thaw cycles in cold 80% methanol. The lysates were centrifuged to remove precipitated protein, and the supernatants with an internal control (10 ul of sodium 2-oxobutyrate) were evaporated, then re-suspended in 40 ul anhydrous pyridine at 70 ^o^C for 15 min. The samples were added to GC/MS autoinjector vials containing 70 ul of N-(tert-butyldimethylsilyl)-N-methyltrifluoroacetamide (MTBSTFA) derivatization reagent and incubated at 70 ^o^C for 1 h. The samples were analyzed using either an Agilent 6890 or 7890 gas chromatograph coupled to an Agilent 5973 N or 5975 C Mass Selective Detector, respectively. The mass isotopologues were corrected for natural abundance.

### Immunoblot analysis

Total protein from T cells was extracted with SDS lysis buffer (1% SDS, 10 mM HEPES, pH 7.0, 2 mM MgCl_2_, 20 U/mL universal nuclease added protease inhibitor) and quantified with BCA assay (23227, Thermo Fisher). Protein was denatured with Laemmli protein sample buffer with 10% 2-mercaptoethanol by incubating at 95 ^o^C for 10 min. Same amount of denatured protein were subjected to electrophoresis using the standard SDS-PAGE method with 4–15% gel (4568086, Bio-Rad) and then wet-transferred to a 0.45 μm polyvinylidene difluoride membrane. After blocking with 5% non-fat milk, membrane was incubated for 2 h with primary antibodies (1:1,000 dilution with 5% BSA in TBST) at room temperature and for 1 h at room temperature with fluorescent-conjugated secondary antibodies (1:10000 dilution with 5% non-fat milk in TBST). Blots were imaged with Licor Odyssey and analyzed with Image Studio Lite.

### CUT&RUN (Cleavage Under Targets and Release Using Nuclease) PCR

CUT&RUN was performed with CUT&RUN Assay Kit (Cell Signaling Technology #86652) according to the manufacturer’s protocol. Briefly, 1 × 10^6^ CD8^+^ T cells from OT-1 mice were captured by Concanavalin A-coated magnetic beads (10 μL per million cells) at room temperature for 5 min with rotation. Captured cells were permeabilized in 100 μL Antibody Binding Buffer (+ Spermidine + Protease inhibitor cocktail + digitonin). After permeabilization, 2 μL H3K27ac antibody or 5 μL IgG were added to each reaction for antibody binding (4 ^o^C, 6 h, with rotation). After 3 times of washing, cells were incubated with Protein A and Protein G-fused Micrococcal Nuclease (pAG-MNase) at 4 ^o^C for 1 h with rotation. By adding cold CaCl_2_, pAG-MNase were activated and antibody-associated protein-DNA complex was released at 4 ^o^C for 30 min. Stop solution was then added to the reaction followed by 30 min additional incubation at 37 ^o^C. The released DNA was purified using spin columns (Cell Signaling Technology #86652). *Tcf7* super enhancer were quantified with qPCR. The levels of H3K27ac enrichment of *Tcf7* super enhancer were determined with by ratio to IgG sample after normalization to *Cd3e*.

### Quantification and statistical analysis

Data were analyzed using GraphPad Prism 8.3. Two-way Analysis of variance (ANOVA) was used to analyze the tumor growth data, logrank test was used to analyze mice survival data, unpaired two-tailed t tests were used to analyze other data unless specified. *P* < 0.05 was considered statistically significant.

### Reporting summary

Further information on research design is available in the Nature Research Reporting Summary linked to this article.

## Supplementary information

Supplementary Information

Description of Additional Supplementary Files

Supplementary Data 1

Supplementary Data 2

Supplementary Data 3

Supplementary Data 4

Supplementary Data 5

Supplementary Data 6

Reporting Summary

## Data Availability

All data are available in the main text or the supplementary materials. Source data are provided with this paper. The single cell RNA sequencing data generated in this study have been deposited to Zenodo (10.5281/zenodo.4387066). https://zenodo.org/record/4387066#.Ytl1uoTMKUk Source data are provided with this paper.

## Source data

Source Data

## Code availability

Custom code was not generated for this manuscript. The following publicly available pipelines were utilized for analysis: raw sequencing data were processed using the 10X Genomics Cell Ranger pipeline (https://support.10xgenomics.com/single-cell-gene-expression/software/pipelines/latest/tutorials/gex-analysis-nature-publication), standard preprocessing and quality control of scRNA-seq data was based on Seurat Guided tutorials (https://satijalab.org/seurat/articles/pbmc3k_tutorial.html), and Monocle2 was used to determine the single-cell pseduotime trajectory (http://cole-trapnell-lab.github.io/monocle-release/tutorials/).
