# Supplementary material for: Lactate increases stemness of CD8 + T cells to augment anti-tumor immunity
Source: Nat Commun. 2022 Sep 6;13:4981. doi: 10.1038/s41467-022-32521-8 (PMC9448806; doi:10.1038/s41467-022-32521-8)
Supplement: Supplementary file 1 — Supplementary Information [file 41467_2022_32521_MOESM1_ESM.pdf]

Supplementary Information

Lactate Increases Stemness of CD8+ T Cells to Augment Anti-Tumor Immunity

Qiang Feng[1, †], Zhida Liu[2, †], Xuexin Yu[3, †], Tongyi Huang[1], Jiahui Chen[1], Jian Wang[1], Jonathan Wilhelm[1], Suxin Li[1], Jiwon Song[1], Wei Li[1], Zhichen Sun[1], Baran Sumer[4,*], Bo Li[3,5,*], Yang-Xin Fu[2,5,*], Jinming Gao[1,4,6*]

[1]Department of Pharmacology, Harold C. Simmons Comprehensive Cancer Center, University of Texas Southwestern Medical Center, Dallas, TX 75390, USA.

[2]Department of Pathology, University of Texas Southwestern Medical Center, Dallas, TX 75390, USA

[3]Lyda Hill Department of Bioinformatics, University of Texas Southwestern Medical Center, Dallas, TX 75390, USA

[4]Department of Otolaryngology, University of Texas Southwestern Medical Center, Dallas, TX 75390, USA

[5]Department of Immunology, University of Texas Southwestern Medical Center, Dallas, TX 75390, USA

[6]Department of Cell Biology, University of Texas Southwestern Medical Center, Dallas, TX 75390, USA

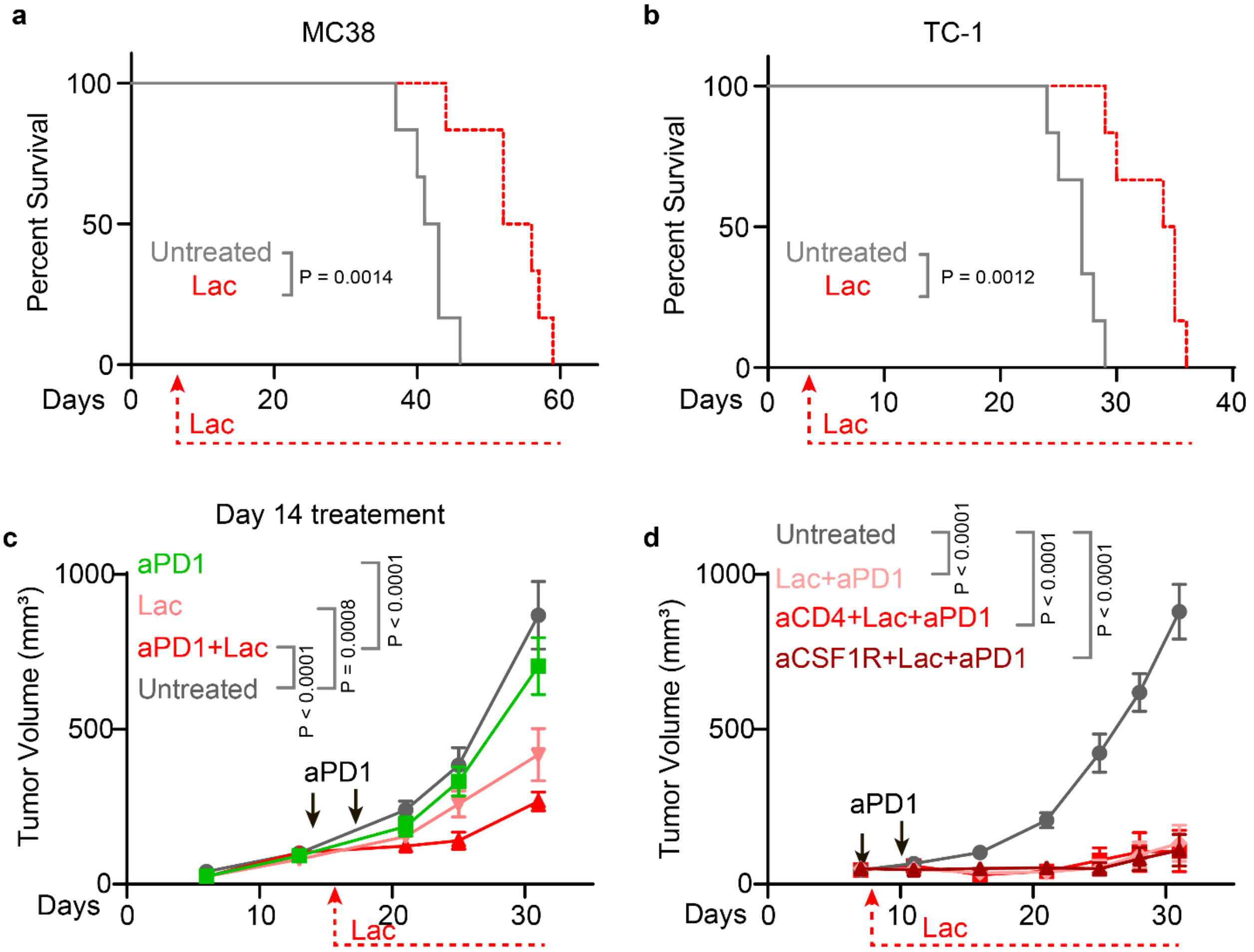

Supplementary Fig. 1. Tumor growth curves of lactate monotherapy and combination therapy with immune cell depletion.

Survival curves of lactate treatment in the MC38 (**a**) and TC-1 (**b**) tumor models. C57BL/6 mice (n = 6) were inoculated with $1{\times}10^6$ MC38 tumor cells and treated by lactate. The dose and treatment regimen of lactate is the same as **Fig. 2b**. The untreated group used the same data in **Fig. 2b** for a side-by-side comparison. C57BL/6 mice (n = 6) were inoculated with $1.5{\times}10^5$ TC-1 tumor cells and treated with lactate. The dose and treatment regimen of lactate is the same as **Fig. 2c**. **c,** Tumor growth curve of MC38 tumor model treated with lactate and anti-PD1 from day 14. C57BL/6 mice (n = 5) were inoculated with $1{\times}10^6$ MC38 tumor cells and treated by lactate. The dose of lactate is the same as **Fig. 2b**. The initial dose of anti-PD1 was on day 14. **d,** $CD4^+$ T and macrophage cell depletion assays in the MC38 tumor model. C57BL/6 mice (n = 6) were inoculated with $1{\times}10^6$ MC38 tumor cells and treated with anti-PD-1 (10 mg/kg, day 7 and 10) in combination with glucose or lactate. Anti-CD4 (10 mg/kg) or anti-CSF1R (20 mg/kg) was

administered from day 3 and then every three days until the end of the experiment. The dose and treatment regimen of lactate and anti-PD-1 are the same as **Fig. 2e**. The untreated and aPD1+Lac group used the same data in **Fig. 1e** for a side-by-side comparison. Data are shown as means ± SEM. P value was determined by one-tail two-way ANOVA with correction using Geisser-Greenhouse method. Source data and summary of all P values are provided in Source Data file.

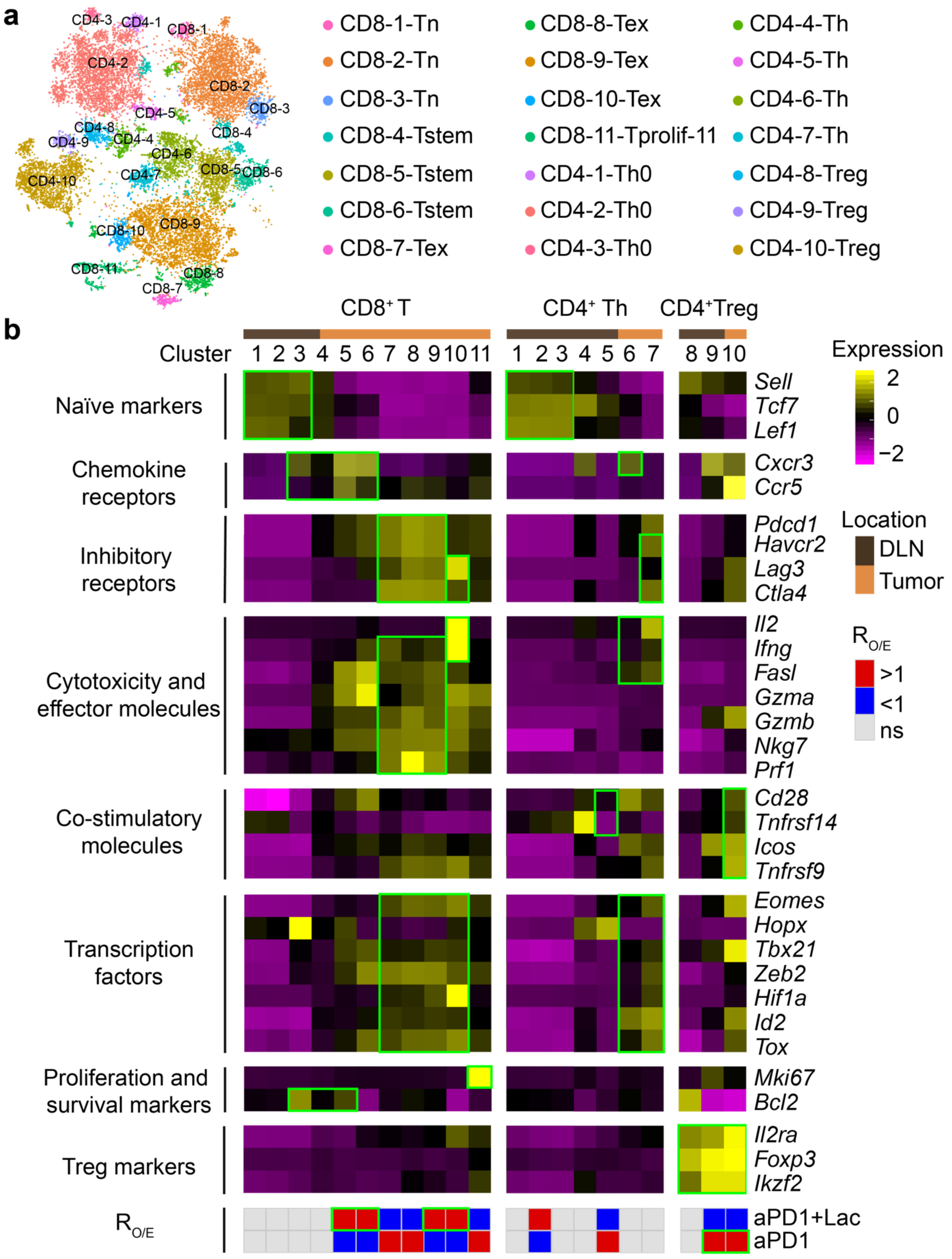
a
CD8-1-Tn
CD8-2-Tn
CD8-3-Tn
CD8-4-Tstem
CD8-5-Tstem
CD8-6-Tstem
CD8-7-Tex
CD8-8-Tex
CD8-9-Tex
CD8-10-Tex
CD8-11-Tprolif-11
CD4-1-Th0
CD4-2-Th0
CD4-3-Th0
CD4-4-Th
CD4-5-Th
CD4-6-Th
CD4-7-Th
CD4-8-Treg
CD4-9-Treg
CD4-10-Treg
b
CD8⁺ T
CD4⁺ Th
CD4⁺Treg
Cluster
Naïve markers
Chemokine receptors
Inhibitory receptors
Cytotoxicity and effector molecules
Co-stimulatory molecules
Transcription factors
Proliferation and survival markers
Treg markers
R O/E
Sell
Tcf7
Lef1
Cxcr3
Ccr5
Pdcd1
Havcr2
Lag3
Ctla4
Il2
Ifng
Fasl
Gzma
Gzmb
Nkg7
Prf1
Cd28
Tnfrsf14
Icos
Tnfrsf9
Eomes
Hopx
Tbx21
Zeb2
Hif1a
Id2
Tox
Mki67
Bcl2
Il2ra
Foxp3
Ikzf2
aPD1+Lac
aPD1
Expression
2
0
−2
Location
DLN
Tumor
>1
<1
ns

Supplementary Fig. 2. Clustering of T cells from single cell RNA sequencing analysis.

a, The tSNE projection of 21, 826 single T cells from 2 treatment groups, showing the formation of 21 main clusters, including 11 for $CD8^+$ T cells, 7 for conventional $CD4^+$ T helper cells (Th: 1-7 of $CD4^+$ clusters) and 3 for regulatory T cells (Treg: 8-10 of $CD4^+$ clusters). Each dot corresponds to one single cell, colored according to cell cluster. **b,** Mean expression of selected T cell function-associated genes in each cell cluster. Green boxes highlight the prominent patterns defining known T cell subtypes. The ratio of observed cell number to expectation ($R_{O/E}$) of Lac effect on anti-PD-1 treatment for each cluster was calculated by one-tail Chi-square test. Source data are provided as a Source Data file.

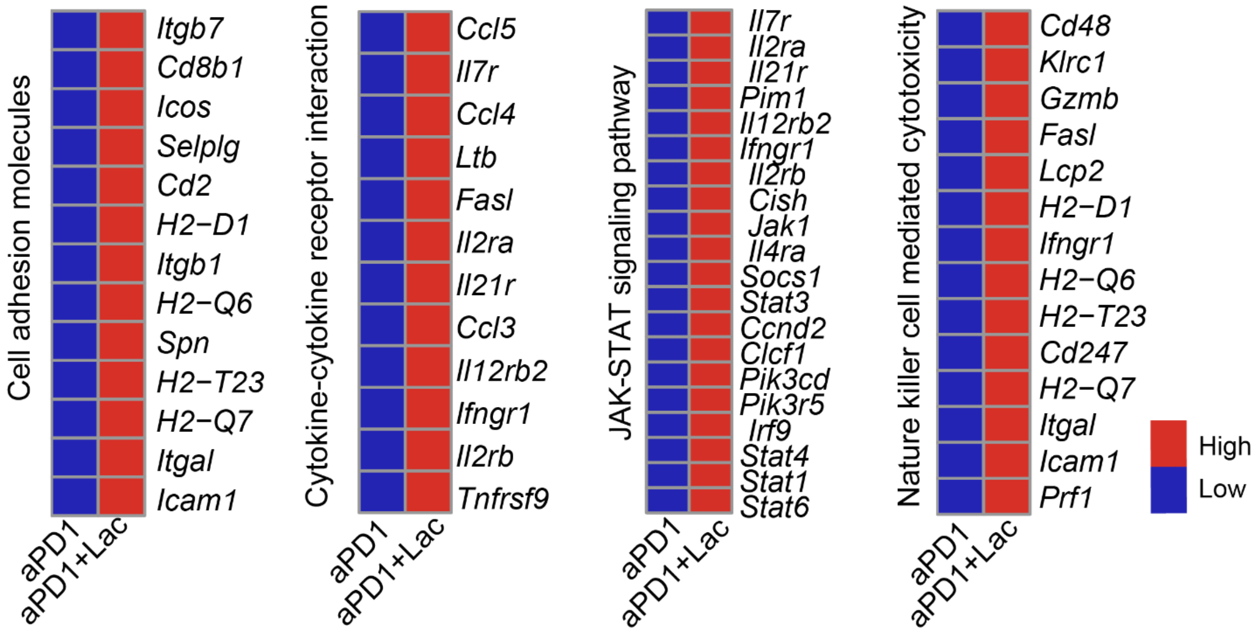

Supplementary Fig. 3. Differentially expressed genes from GSEA analysis in Figure 3. Source data are provided as a Source Data file.

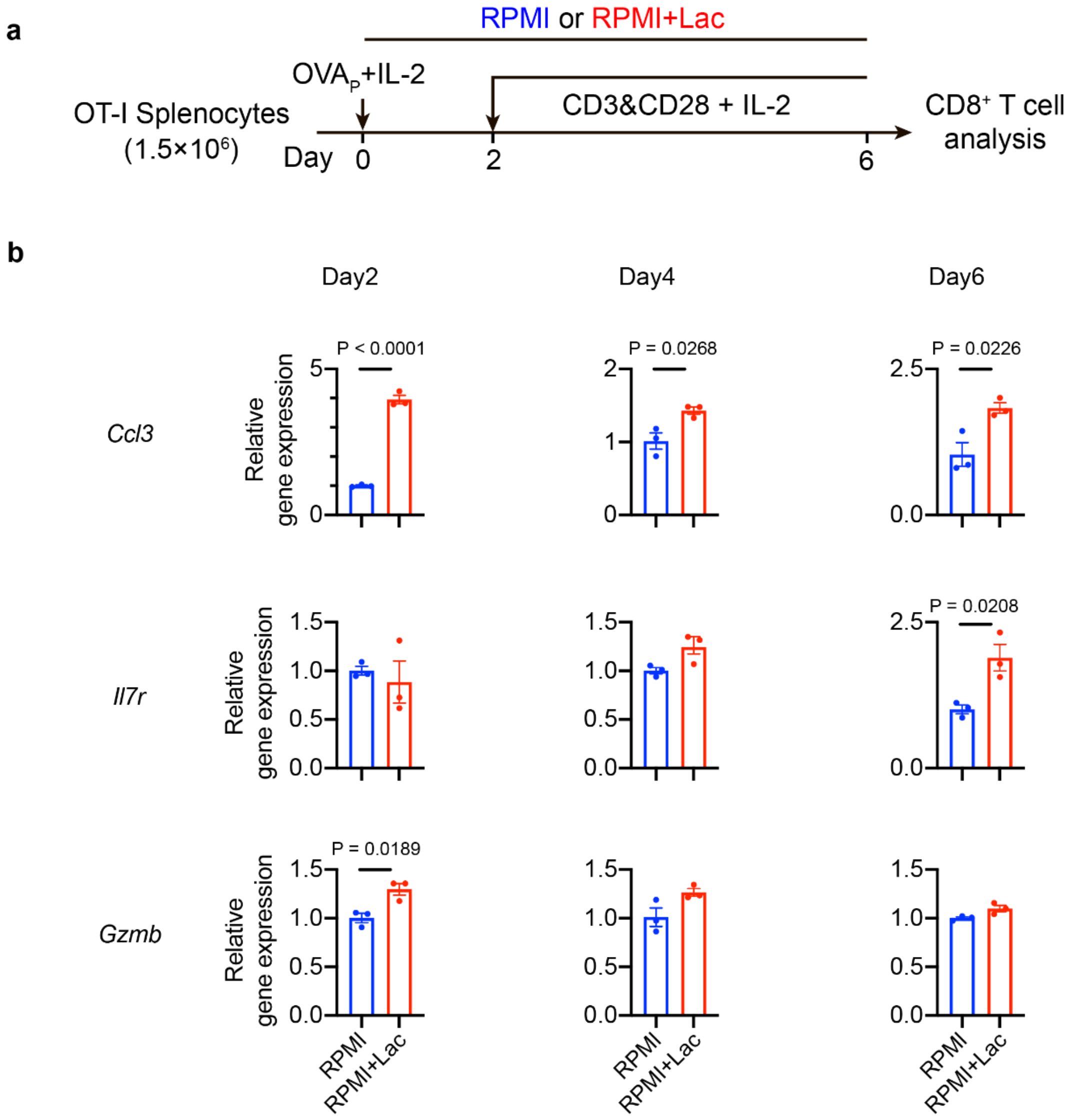

Supplementary Fig. 4. Verification of the expression of genes elevated by lactate treatment from single cell analysis.

a, Schematic illustration of the CD8$^+$ T cell culture assay. **b,** Gene expression quantified with qPCR for *Ccl3*, *Il7r*, and *Gzmb*. Data are shown as means ± SEM. n= 3 biologically independent samples. P value was determined by one-tail unpaired t-test. Source data are provided as a Source Data file.

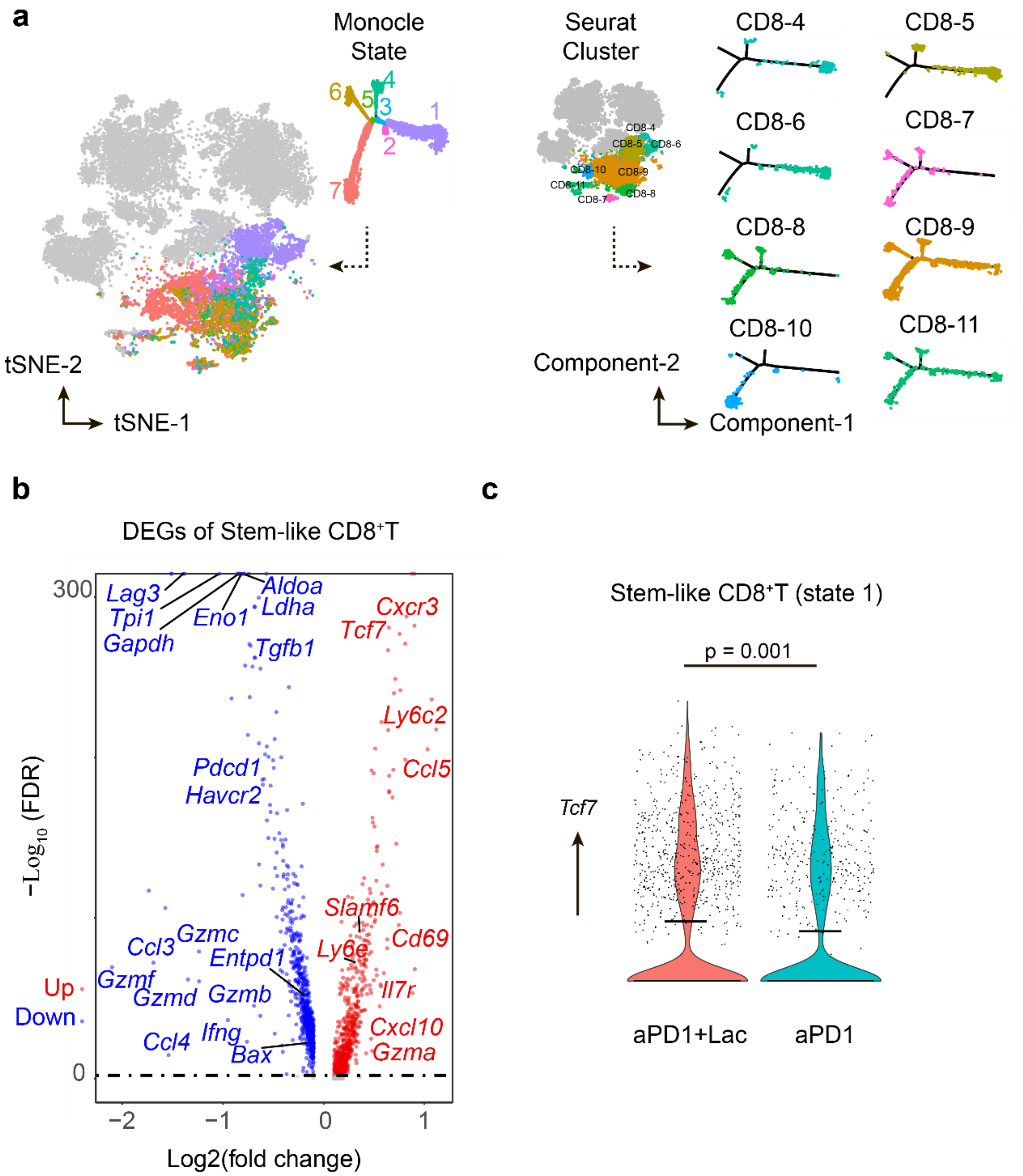

Supplementary Fig. 5. Delineation of stem-like CD8$^+$ T cell population by pseudotime analysis.

a, Correlation of Seurat clusters and monocle states. Stem-like T cells in state 1 are mostly from Seurat cluster CD8-4, CD8-5, CD8-6 and part of CD8-9. **b,** Volcano plot of differentially expressed genes by stem-like CD8$^+$ T cells. **c,** *Tcf7* gene expression in stem-like CD8$^+$ T cells. p value was determined by two-tail Mann Whitney U test. Source data are provided as a Source Data file.

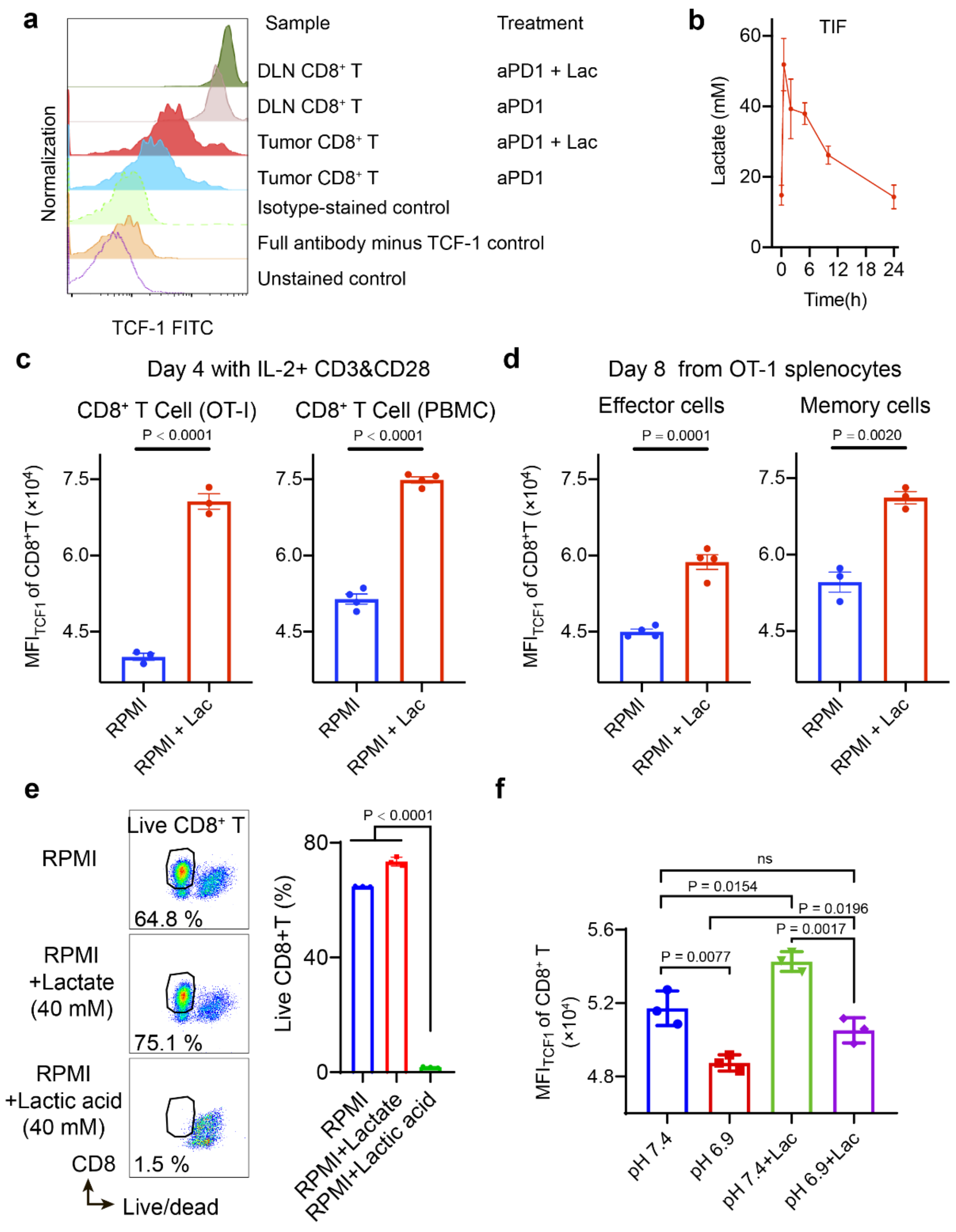

Supplementary Fig. 6. Lactate increases the expression of TCF-1 *in vivo* and *ex vivo* while lactic acid induces T cell death.

a, Flow cytometry histograms of TCF-1 expressions on $CD8^+$ T cells under different treatment conditions. Unstained control, full antibody minus anti-TCF-1 and isotype IgG were used as controls. **b,** Lactate concentration in tumor interstitial fluids (TIF) after subcutaneous lactate injection (n = 3 biologically independent samples). **c,** Quantification of TCF-1 expressions in *ex*

vivo cultured $CD8^+$ T cells from OT-I mice or human PBMCs on day 4 (n = 3 biologically independent samples for OT-I cell, n = 4 biologically independent samples for PBMC). **d,** Quantification of TCF-1 expressions in *ex vivo* cultured effector or memory $CD8^+$ T cells on day 8. For memory T cells induction, $CD8^+$ T cells from OT-1 splenocytes were activated with OVAp for two days and purified with ficoll, followed by 6-Day culture with addition of IL-15 (20 ng/mL) (n = 4 biologically independent samples for effector cell culture, n = 3 biologically independent samples for memory cell culture). **e,** Flow cytometry plot and quantification of live $CD8^+$ T cells from OT-I mice in RPMI medium with sodium lactate (pH 7.4) or lactic acid (pH 4.6) after two days (n = 3 biologically independent samples). **f,** Quantification of TCF-1 expression in $CD8^+$ T cells cultured in medium with mildly acidic pH and lactate concentrations (n = 3 biologically independent samples). Data are shown as means ± SEM. P value was determined by two-tail unpaired t-test (**c**, **d**) or one-tail one-way ANOVA (**e**, **f**). Source data are provided as a Source Data file.

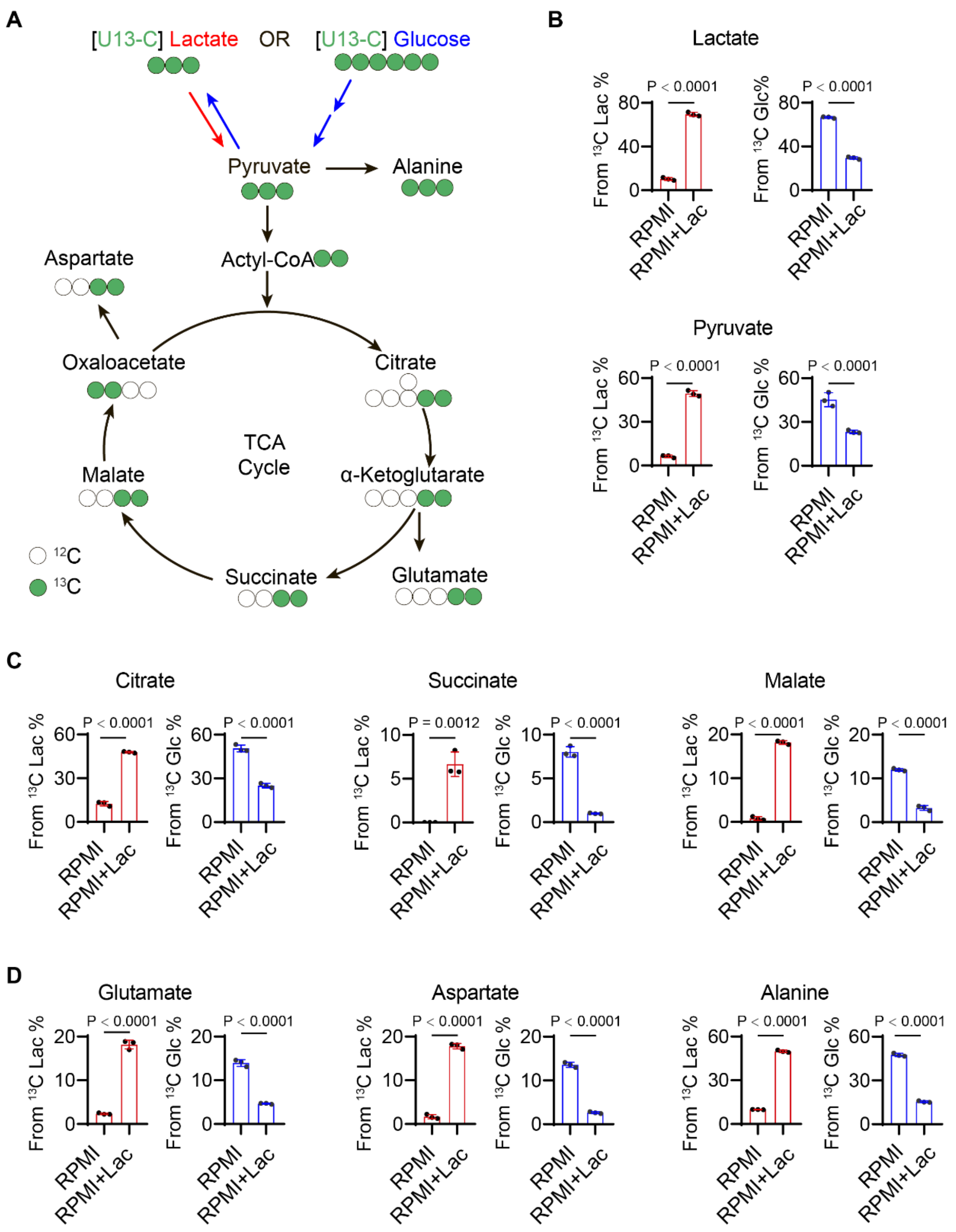

Supplementary Fig. 7. Lactate feeds TCA cycle during *ex vivo* culture of CD8^{+} T cell.

a, Schematic illustration of isotopologues generated from ^{13}C-glucose or ^{13}C-lactate. Normalized abundance of intracellular lactate and pyruvate (**b**), TCA cycle intermediates (**c**) and derived amino acids (**d**). Data are shown as means ± SEM. n = 3 biologically independent samples. Source data are provided as a Source Data file.

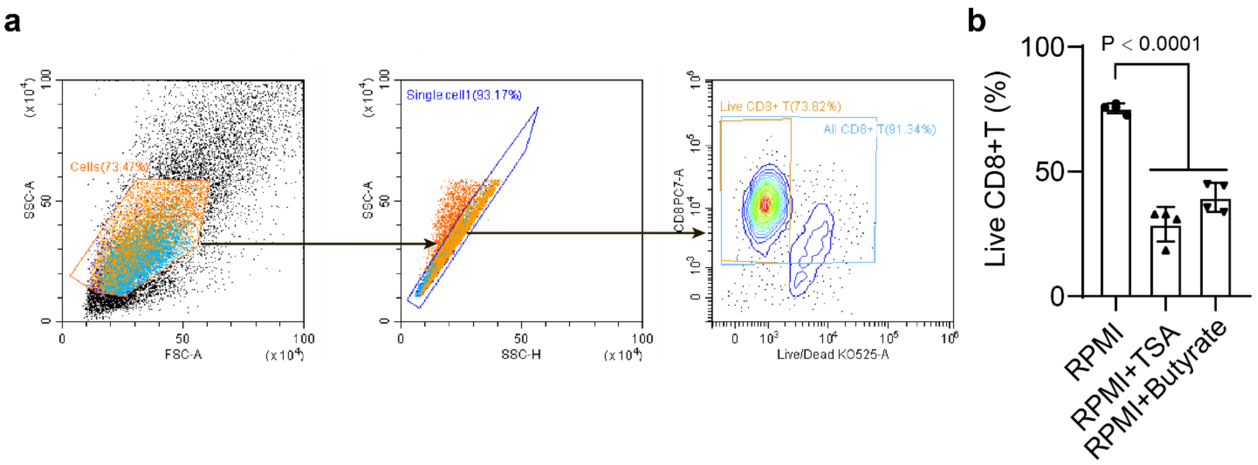

Supplementary Fig. 8. Cell viability of CD8+ T cell cultured with TSA or butyrate after 4-day culture.

a, Gating strategy for quantification of live CD8+ T cells. CD8+ T cells from OT-I mice were cultured with different drug for 4 days (TSA = 1 nM, Butyrate = 250 μM). **b**, Statistical analysis of live CD8+ T cells with different treatments. n = 4 biologically independent samples. Data are shown as means ± SD. P value was determined by one-way ANOVA and corrected by Tukey method for multiple comparisons. Source data are provided as a Source Data file.

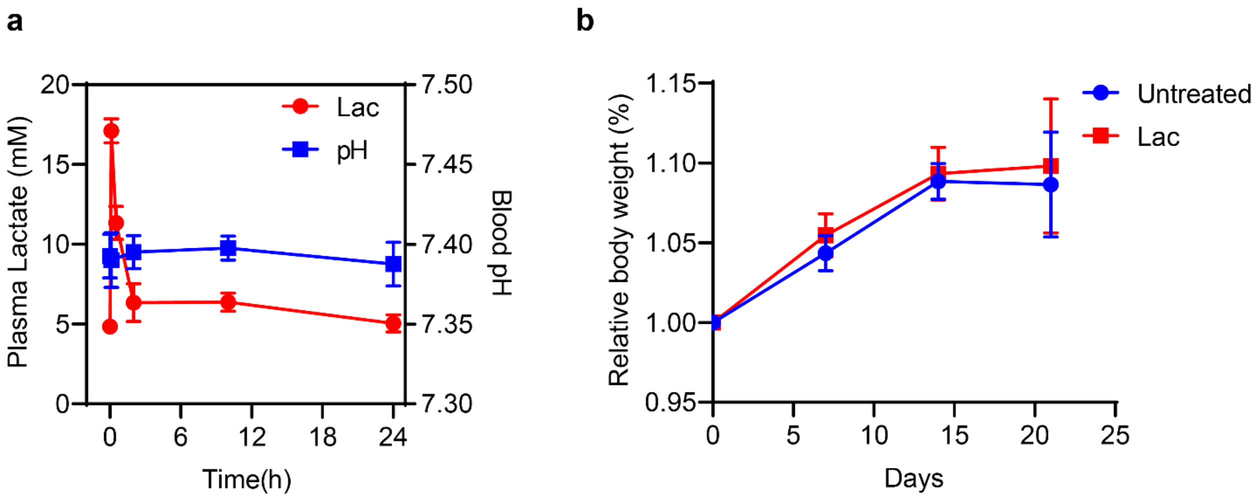

Supplementary Fig. 9. Effect of subcutaneously injected lactate solution on blood lactate concentrations and pH levels and body weight.

a, Lactate concentration and pH in blood as a function of time after subcutaneous lactate injection (1.68 g/kg). (For lactate, n = 3 biologically independent samples, for pH, n = 4 biologically independent samples). **b**, Change of body weight after daily lactate treatment from Day 0 to 21. (n = 5 biologically independent samples). Data are shown as means ± SEM. Source data are provided as a Source Data file.

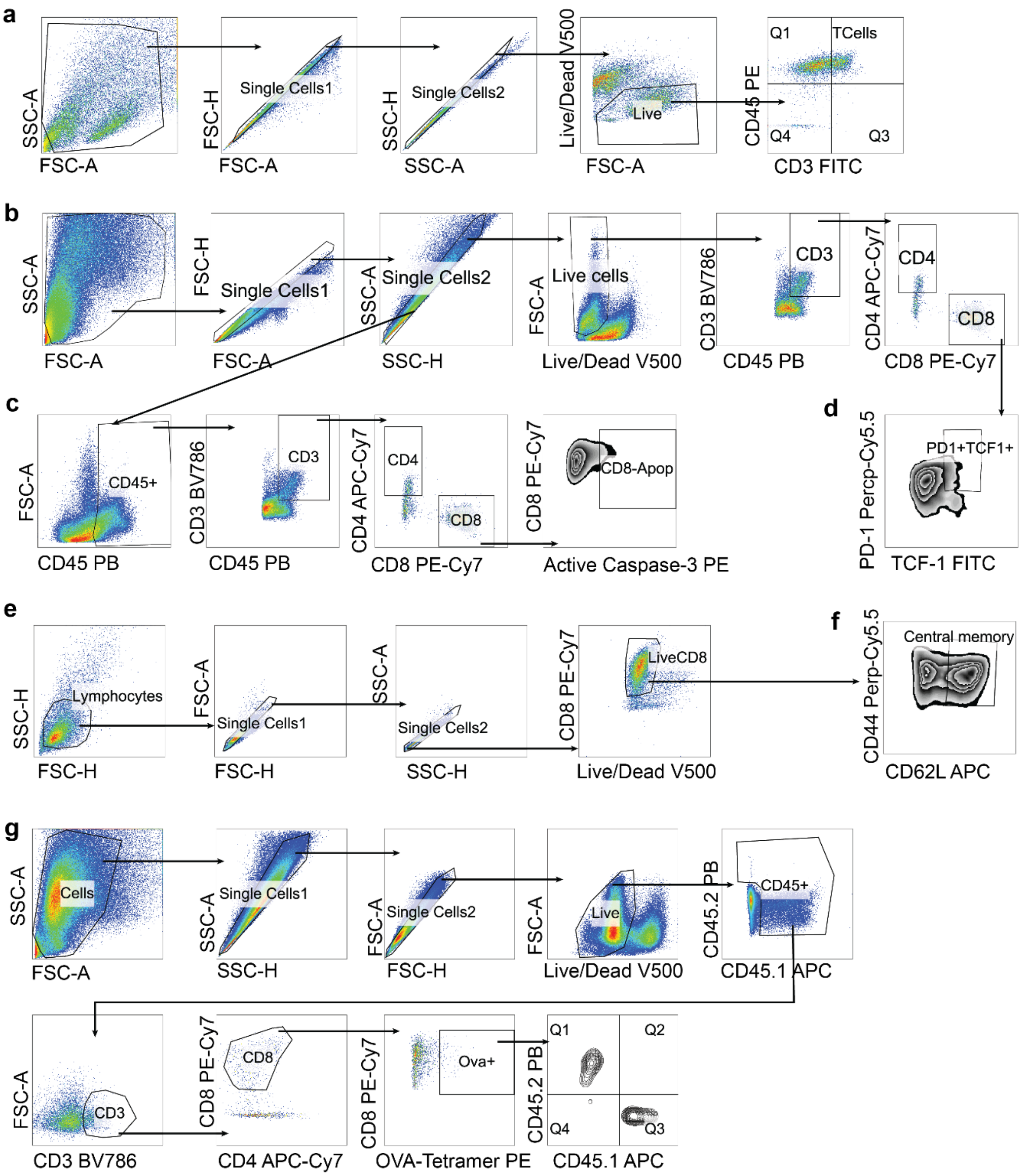

Supplementary Fig. 10. Gating strategies used for cell sorting and flow cytometry analysis.

a, Gating strategy to sort tumor infiltrating T cells for single cell analysis. Data from sorted cells were used in Fig 3b-d, Fig 4a-b, Supplementary Fig 2, 3, 5. **b**, Gating strategy for analyzing tumor infiltrating CD8$^+$ T cells number and TCF-1 expression in Fig. 2e, Fig. 3e,f, supplementary Fig. 6a. **c,** Gating strategy for analyzing apoptotic tumor infiltrating CD8$^+$ T cells in Fig. 2f. **d,** Gating

strategy for analyzing tumor infiltrating stem-like $CD8^+$ T cells number in Fig. 4d. **e,** Gating strategy for analyzing $CD8^+$ T cells in cell culture in Fig. 5 b-e, h, i, 6d, supplementary Fig. 6 c, e, f. **f,** Gating strategy for analyzing IL-15 induced memory $CD8^+$ T cells in cell culture in supplementary Fig. 6d. g, Gating strategy for analyzing tumor infiltrating ova-targeting $CD8^+$ T cells from recipients or donor in Fig. 7d-f.
