# Supplementary material for: Lactate increases stemness of CD8 + T cells to augment anti-tumor immunity
Source: Nat Commun. 2022 Sep 6;13:4981. doi: 10.1038/s41467-022-32521-8 (PMC9448806; doi:10.1038/s41467-022-32521-8)
Supplement: Supplementary file 2 — Description of Additional Supplementary Files [file 41467_2022_32521_MOESM2_ESM.pdf]

Description of Additional Supplementary Files

File Name: Supplementary Data 1
Description: Cell count in each cluster from single cell RNA sequencing.

File Name: Supplementary Data 2
Description: Marker gene list for different clusters in CD3 population.

File Name: Supplementary Data 3
Description: Marker gene list for stem-like CD8+ T cells.

File Name: Supplementary Data 4
Description: Untargeted metabolomics data for CD8+ T cell treated with RPMI or RPMI+Lactate.

File Name: Supplementary Data 5
Description: Stable isotope tracing with 13C-lactate and 13C-Glucose in CD8+ T cell.

File Name: Supplementary Data 6
Description: Key resource table.
