# Supplementary material for: Lactate increases stemness of CD8 + T cells to augment anti-tumor immunity
Source: Nat Commun. 2022 Sep 6;13:4981. doi: 10.1038/s41467-022-32521-8 (PMC9448806; doi:10.1038/s41467-022-32521-8)
Supplement: Supplementary file 9 — Reporting Summary [file 41467_2022_32521_MOESM9_ESM.pdf]

nature research

Corresponding author(s): Jinming Gao, Bo Li, Yang-Xin Fu

Last updated by author(s): Jun 28, 2022

Reporting Summary

Nature Research wishes to improve the reproducibility of the work that we publish. This form provides structure for consistency and transparency in reporting. For further information on Nature Research policies, see our Editorial Policies and the Editorial Policy Checklist.

Statistics

For all statistical analyses, confirm that the following items are present in the figure legend, table legend, main text, or Methods section.

n/a	Confirmed	
☐	☒	The exact sample size (n) for each experimental group/condition, given as a discrete number and unit of measurement
☐	☒	A statement on whether measurements were taken from distinct samples or whether the same sample was measured repeatedly
☐	☒	The statistical test(s) used AND whether they are one- or two-sided<br>*Only common tests should be described solely by name; describe more complex techniques in the Methods section.*
☒	☐	A description of all covariates tested
☐	☒	A description of any assumptions or corrections, such as tests of normality and adjustment for multiple comparisons
☐	☒	A full description of the statistical parameters including central tendency (e.g. means) or other basic estimates (e.g. regression coefficient) AND variation (e.g. standard deviation) or associated estimates of uncertainty (e.g. confidence intervals)
☐	☒	For null hypothesis testing, the test statistic (e.g. F, t, r) with confidence intervals, effect sizes, degrees of freedom and P value noted<br>*Give P values as exact values whenever suitable.*
☒	☐	For Bayesian analysis, information on the choice of priors and Markov chain Monte Carlo settings
☒	☐	For hierarchical and complex designs, identification of the appropriate level for tests and full reporting of outcomes
☒	☐	Estimates of effect sizes (e.g. Cohen's d, Pearson's r), indicating how they were calculated

Our web collection on statistics for biologists contains articles on many of the points above.

Software and code

Policy information about availability of computer code

Data collection	CytExpert2.4.0.28 (Beckman counter) ; NovaSeq S4(Illumina); FACSDiva9.0.1 (BD biosciences)
Data analysis	GraphPad Prism software 8.3; FlowJo10.4; R(3.6.1&4.0.1); Seurat 3.0.1; ClusterProfiler 3.12; Monocle-2; Metaboanalyst 5.0; Limma3.40.5

For manuscripts utilizing custom algorithms or software that are central to the research but not yet described in published literature, software must be made available to editors and reviewers. We strongly encourage code deposition in a community repository (e.g. GitHub). See the Nature Research guidelines for submitting code & software for further information.

Data

Policy information about availability of data

All manuscripts must include a data availability statement. This statement should provide the following information, where applicable:
- Accession codes, unique identifiers, or web links for publicly available datasets
- A list of figures that have associated raw data
- A description of any restrictions on data availability

All data are available in the main text or the supplementary materials. Source data are provided with this paper. The single cell RNA sequencing data generated in this study have been deposited to Zenodo (DOI: 10.5281/zenodo.4387066).

Field-specific reporting

Please select the one below that is the best fit for your research. If you are not sure, read the appropriate sections before making your selection.

☒ Life sciences ☐ Behavioural & social sciences ☐ Ecological, evolutionary & environmental sciences

For a reference copy of the document with all sections, see nature.com/documents/nr-reporting-summary-flat.pdf

Life sciences study design

All studies must disclose on these points even when the disclosure is negative.

Sample size	Sample size was determined based on previous publications containing similar procedure to maintain the balance between reaching the statistical significance and minimizing the number of animal/reagents use. Published papers was used as references (PMID:33338425; 32231300; 31185213).
Data exclusions	No sample exclusion.
Replication	All experiments and in vitro assays were repeated in at least two independent experiments. Single cell transcriptomics and metabolomics were from one experiments and key discoveries were validated with other assays.
Randomization	All samples were randomly allocated to experimental groups.
Blinding	No blinding. Blinding was not needed in the study because conditions were well controlled with syngenic mice. Blinding is not typically used in the field.

Reporting for specific materials, systems and methods

We require information from authors about some types of materials, experimental systems and methods used in many studies. Here, indicate whether each material, system or method listed is relevant to your study. If you are not sure if a list item applies to your research, read the appropriate section before selecting a response.

Materials & experimental systems

n/a	Involved in the study	
☐	☒	Antibodies
☐	☒	Eukaryotic cell lines
☒	☐	Palaeontology and archaeology
☐	☒	Animals and other organisms
☐	☒	Human research participants
☒	☐	Clinical data
☒	☐	Dual use research of concern

Methods

n/a	Involved in the study	
☒	☐	ChIP-seq
☐	☒	Flow cytometry
☒	☐	MRI-based neuroimaging

Antibodies

Antibodies used

InVivoPlus anti-mouse CD4 (GK1.5,BioXcell, Cat# BP0003-1)
InVivoPlus anti-mouse CD8α (53-6.7, BioXcell, Cat# BP0004-1)
InVivoPlus anti-mouse CSF1R (AFS98, BioXcell, Cat# BP0213)
InVivoPlus anti-mouse PD-1 (29F.1A12, BioXcell, Cat# BP0273)
Anti-mouse CD45 (Cell sorting, REA737, Miltenyi, Cat# 130-110-797)
Anti-mouse CD3 (Cell sorting, 17A2,biolegend, Cat# 100203)
Anti-mouse CD45 (Flow cytometry, 30-F11, biolegend, Cat# 103126)
Anti-mouse CD3Ɛ (Flow cytometry, 145-2C11, biolegend, Cat# 100355)
Anti-mouse CD8α (Flow cytometry, 53-6.7, biolegend, Cat# 100721)
Anti- active Caspase-3 (Flow cytometry, C92-605, BD, Cat# 550821)
Anti-mouse PD-1 (Flow cytometry, 29F.1A12, biolegend, Cat# 135207)
Anti-mouse Lag-3 (Flow cytometry, C9B7W, biolegend, Cat# 125209)
Anti-TCF1/TCF7 (Flow cytometry, C63D9, Cell signaling Technology, Cat# 6444S)
Anti-mouse CXCR3 (Flow cytometry, CXCR3-173, biolegend, Cat# 126511)
Anti-human CD8 (Flow cytometry, SK1, biolegend, Cat# 344711)
Anti-human CXCR3 (Flow cytometry, G025H7, Biolegend, Cat# 353707)
Anti-mouse CD45.1 (Flow cytometry, A20, biolegend, Cat# 110713)
Anti-mouse CD45.2 (Flow cytometry, 104, biolegend, Cat# 109819)
Anti-mouse CD3 (Cell activation, 17A2, biolegend, Cat# 100239)
Anti-mouse CD28 (Cell activation, 37.51, Biolegend, Cat# 102115)
iTAg Tetramer/PE - H-2 Kb OVA (SIINFEKL, invivogen, Cat# vac-sin)

Histone H3 Antibody (Western Blot, Cell signaling Technology, Cat# 9715S)
Histone H3K27ac antibody (mAb) (CUT&RUN, WB, Active Motif, Cat# 39085)
Rabbit (DA1E) mAb IgG XP® Isotype Control (CUT&RUN, Cell signaling Technology, Cat# 66362S)
Anti-rabbit IgG (H+L) (DyLight™ 800 4X PEG Conjugate) (Cell Signaling Technology, Cat# 5151P)
Anti-mouse IgG (H+L) (DyLight™ 680 Conjugate)(Cell Signaling Technology, Cat# 5470P)

Validation

All antibodies are from commercial companies and are well validated by manufacturer and widely used in researches. Their validation data are available on the manufacturer's websites:
InVivoPlus anti-mouse CD4 (GK1.5,BioXcell, Cat# BP0003-1):
https://bxcell.com/product/invivoplus-anti-m-cd4/

InVivoPlus anti-mouse CD8α (53-6.7, BioXcell, Cat# BP0004-1):
https://bxcell.com/product/invivoplus-anti-m-cd8a/

InVivoPlus anti-mouse CSF1R (AFS98, BioXcell, Cat# BE0213):
https://bxcell.com/product/invivoplus-anti-mouse-csf1r-cd115/

InVivoPlus anti-mouse PD-1 (29F.1A12, BioXcell, Cat# BP0273):
https://bxcell.com/product/invivoplus-anti-mouse-pd-1-cd279/

Anti-mouse CD45 (Cell sorting, REA737, Miltenyi, Cat# 130-110-797):
https://www.miltenyibiotec.com/US-en/products/cd45-antibody-anti-mouse-reafinity-rea737.html#vioblue:30-ug-in-200-ul

Anti-mouse CD3 (Cell sorting, 17A2,biolegend, Cat# 100203):
https://www.biolegend.com/en-us/products/fitc-anti-mouse-cd3-antibody-45

Anti-mouse CD45 (Flow cytometry, 30-F11, biolegend, Cat# 103126):
https://www.biolegend.com/en-us/products/pacific-blue-anti-mouse-cd45-antibody-3102

Anti-mouse CD3Ɛ (Flow cytometry, 145-2C11, biolegend, Cat# 100355):
https://www.biolegend.com/en-us/products/brilliant-violet-785-anti-mouse-cd3epsilon-antibody-12081

Anti-mouse CD8α (Flow cytometry, 53-6.7, biolegend, Cat# 100721):
https://www.biolegend.com/en-us/products/pe-cyanine7-anti-mouse-cd8a-antibody-1906

Anti- active Caspase-3 (Flow cytometry, C92-605, BD, Cat# 550821):
https://www.bdbiosciences.com/en-us/products/reagents/flow-cytometry-reagents/research-reagents/single-color-antibodies-ruo/pe-rabbit-anti-active-caspase-3.550821

Anti-mouse PD-1 (Flow cytometry, 29F.1A12, biolegend, Cat# 135207):
https://www.biolegend.com/en-us/products/percp-cyanine5-5-anti-mouse-cd279-pd-1-antibody-6496

Anti-TCF1/TCF7 (Flow cytometry, C63D9, Cell signaling Technology, Cat# 6444S):
https://www.cellsignal.com/products/antibody-conjugates/tcf1-tcf7-c63d9-rabbit-mab-alexa-fluor-488-conjugate/6444?site-search-type=Products&N=4294956287&Ntt=6444s&fromPage=plp&_requestid=29298

Anti-mouse CXCR3 (Flow cytometry, CXCR3-173, biolegend, Cat# 126511):
https://www.biolegend.com/en-us/products/apc-anti-mouse-cd183-cxcr3-antibody-4683

Anti-human CD8 (Flow cytometry, SK1, biolegend, Cat# 344711):
https://www.biolegend.com/en-us/products/pe-cyanine7-anti-human-cd8-antibody-6390

Anti-human CXCR3 (Flow cytometry, G025H7, Biolegend, Cat# 353707):
https://www.biolegend.com/en-us/products/apc-anti-human-cd183-cxcr3-antibody-7580

Anti-mouse CD45.1 (Flow cytometry, A20, biolegend, Cat# 110713):
https://www.biolegend.com/en-us/products/apc-anti-mouse-cd45-1-antibody-2319

Anti-mouse CD45.2 (Flow cytometry, 104, biolegend, Cat# 109819):
https://www.biolegend.com/en-us/products/pacific-blue-anti-mouse-cd45-2-antibody-3108

Anti-mouse CD3 (Cell activation, 17A2, biolegend, Cat# 100239):
https://www.biolegend.com/en-us/products/ultra-leaf-purified-anti-mouse-cd3-antibody-8078

Anti-mouse CD28 (Cell activation, 37.51, Biolegend, Cat# 102115):
https://www.biolegend.com/en-us/products/ultra-leaf-purified-anti-mouse-cd28-antibody-7733

iTAg Tetramer/PE - H-2 Kb OVA (SIINFEKL, MBL, Cat# TB-5001-1):
https://www.mblintl.com/products/tb-5001-1/

Histone H3 Antibody (Western Blot, Cell signaling Technology, Cat# 9715S):
https://www.cellsignal.com/products/primary-antibodies/histone-h3-antibody/9715?site-search-type=Products&N=4294956287&Ntt=9715s&fromPage=plp&_requestid=29798

Histone H3K27ac antibody (mAb) (CUT&RUN, WB, Active Motif, Cat# 39085):
https://www.activemotif.com/catalog/details/39685/histone-h3-acetyl-lys27-antibody-mab-clone-mabi-0309

Rabbit (DA1E) mAb IgG XP® Isotype Control (CUT&RUN, Cell signaling Technology, Cat# 66362S): https://www.cellsignal.com/products/primary-antibodies/rabbit-da1e-mab-igg-xp-isotype-control-cut-amp-run/66362?site-search-type=Products&N=4294956287&Ntt=66362s&fromPage=plp&_requestid=29849

Anti-rabbit IgG (H+L) (DyLight™ 800 4X PEG Conjugate) (Cell Signaling Technology, Cat# 5151P): https://www.cellsignal.com/products/secondary-antibodies/anti-rabbit-igg-h-l-dylight-800-4x-peg-conjugate/5151?site-search-type=Products&N=4294956287&Ntt=5151p&fromPage=plp&_requestid=29885

Anti-mouse IgG (H+L) (DyLight™ 680 Conjugate)(Cell Signaling Technology, Cat# 5470P): https://www.cellsignal.com/products/secondary-antibodies/anti-mouse-igg-h-l-dylight-680-conjugate/5470?site-search-type=Products&N=4294956287&Ntt=5470p&fromPage=plp&_requestid=29924

Eukaryotic cell lines

Policy information about cell lines

Cell line source(s)

Murine melanoma B16F10 and colon adenocarcinoma MC38 cell lines were purchased from American Type Culture Collection (ATCC). TC-1 is a tumor cell line transformed from C57BL/6 primary mouse lung epithelial cells and expressing HPV16 E6, E7 and c-Ha-ras oncoproteins. This cell line is generated and kindly provided by Dr. T.C. Wu at Johns Hopkins University.

Authentication

No further authentication was performed before use.

Mycoplasma contamination

All cell line used were tested Mycoplasma-free before use.

Commonly misidentified lines
(See ICLAC register)

No commonly misidentified cell line was used.

Palaeontology and Archaeology

Specimen provenance

Provide provenance information for specimens and describe permits that were obtained for the work (including the name of the issuing authority, the date of issue, and any identifying information).

Specimen deposition

Indicate where the specimens have been deposited to permit free access by other researchers.

Dating methods

If new dates are provided, describe how they were obtained (e.g. collection, storage, sample pretreatment and measurement), where they were obtained (i.e. lab name), the calibration program and the protocol for quality assurance OR state that no new dates are provided.

☐ Tick this box to confirm that the raw and calibrated dates are available in the paper or in Supplementary Information.

Ethics oversight

Identify the organization(s) that approved or provided guidance on the study protocol, OR state that no ethical approval or guidance was required and explain why not.

Note that full information on the approval of the study protocol must also be provided in the manuscript.

Animals and other organisms

Policy information about studies involving animals; ARRIVE guidelines recommended for reporting animal research

Laboratory animals

6-10 weeks. Female or male C57BL/6J(Strain #:000664), NOD.Cg-Prkdcscid/J(Strain #:001303), C57BL/6-Tg(TcraTcrb)1100Mjb/J (Strain #:003831), B6.129S7-Rag1tm1Mom/J (Strain #:002216), B6.SJL-PtprcaPepcb/BoyJ (Strain #:002014) mice were purchased from Jackson Laboratory.

Wild animals

No wild animal was used.

Field-collected samples

No field-collected sample was used.

Ethics oversight

All animal experiments were performed with ethical compliance and approval from Institutional Animal Care and Use Committee of the University of Texas Southwestern Medical Center.

Note that full information on the approval of the study protocol must also be provided in the manuscript.

Human research participants

Policy information about studies involving human research participants

Population characteristics

Sterile blood was obtained at the time of cesarean section from de-identified human umbilical cords that are normally discarded. To maintain anonymity, links between the donor's medical and social histories including fetal sex are not maintained.

Recruitment	The cord blood were from random de-identified patients. No potential self-selection bias.
Ethics oversight	Human cord blood samples were obtained from UT Southwestern (UTSW) Parkland Hospital in compliance to the regulation and the use approval of human cord blood at UTSW medical center(STU 112010-047). The procedure is approved through a protocol exempt from informed consent as approved by the Institutional Review Board of UTSW and the Office for Human Research Protections (OHRP) supported by the U.S. Department of Health and Human Services.

Note that full information on the approval of the study protocol must also be provided in the manuscript.

Flow Cytometry

Plots

Confirm that:

☒ The axis labels state the marker and fluorochrome used (e.g. CD4-FITC).

☒ The axis scales are clearly visible. Include numbers along axes only for bottom left plot of group (a 'group' is an analysis of identical markers).

☒ All plots are contour plots with outliers or pseudocolor plots.

☒ A numerical value for number of cells or percentage (with statistics) is provided.

Methodology

Sample preparation	Single cell suspensions were obtained from cell culture or mouse tissues. Mouse tumors were dissociated by Collagenase (1 mg/mL) and DNAse I (0.2 mg/mL) and lymph nodes were dissociated with 70 µm cell strainer. For each single cell suspension, Fc receptor was blocked with anti-FcγIII/II (clone 2.4G2) for 20 minutes, followed by staining with selective antibodies of cell surface markers and live/dead dyes. Intracellular markers including active caspase 3 and TCF-1 were stained after cell permeabilization with True-Nuclear transcription factor buffer set (BioLegend). Data were collected on BD LSR Fortessa or Beckman CytoFLEX flow cytometer and analyzed by FlowJo (Tree Star Inc., Ashland, OR) software.
Instrument	cytoFLEX (Backman coulter),BD LSRFortessa
Software	CytExpert2.4.0.28, FlowJo software v9.3.2, BD FACSDiva v9.0.1,
Cell population abundance	For in vitro analysis, at least 5000 live CD8 T cell were collected for analysis. For in vivo analysis, at least 20000 live CD45 Cell were collected for analysis.
Gating strategy	Physical parameter and fixable viability Dye eFlour 506 (eBioscience) was used to exclude the dead cells. Positive populations were defined using not stained cells as reference. Isotype controls were used to confirm the specificity of the staining

☒ Tick this box to confirm that a figure exemplifying the gating strategy is provided in the Supplementary Information.
